# Systematic Analysis of Molecular Subtypes and Immune Prediction Based on CD8 T Cell Pattern Genes Based on Head and Neck Cancer

**DOI:** 10.1155/2022/1500493

**Published:** 2022-08-25

**Authors:** Li Yanwei, Feng He, Shuang Liu, Zhanyu Pan

**Affiliations:** ^1^Academy of Medical Engineering and Translational Medicine, Tianjin, China; ^2^Tianjin Key Laboratory of Brain Science and Neural Engineering, Tianjin University, Tianjin, China; ^3^Department of Integrative Oncology, Tianjin Medical University Cancer Institute, Hospital and Key Laboratory of Cancer Prevention and Therapy, Tianjin, China

## Abstract

CD8^+^ T lymphocytes, also known as cytotoxic T lymphocytes, are the most powerful antitumour cells in the human body. Patients with head and neck squamous cell carcinoma (HNSCC) in whom CD8^+^ T lymphocyte infiltration is high have a better prognosis. However, the clinical significance and prognostic significance of CD8^+^ T cell-related regulatory genes in HNSCC remain unclear, and further research is required. In total, 446 CD8^+^ T cell-related genes were obtained using WGCNA. It was discovered that 111 genes included within the TCGA and GSE65858 datasets were intimately linked to the patient's prognosis. These genes were included in the subsequent analysis. According to consensus clustering analysis, HNSCC samples were classified into 3 subtypes (IC1, IC2, and IC3). There were substantial differences between the three subtypes in terms of immunological molecules, immune function, and the response to drug treatment. In addition, the 8-gene signature, which was generated premised on CD8^+^ T cell-related genes, exhibited stable prognostic prediction in the TCGA and GEO datasets and different HNSCC patient subgroups and independently served as a prognostic indicator for HNSCC. More importantly, the 8-gene signature effectively predicted immunotherapy response. We first constructed a molecular subtype of HNSCC based on CD8^+^ T cell-related genes. Between the three subtypes, there were significant differences in the prognosis, clinical features, immunological molecules, and drug treatment response. The 8-gene signature that was further constructed effectively predicted prognosis and immunotherapy response.

## 1. Introduction

Head and neck squamous cell carcinoma (HNSCC) accounts for roughly 95 percent of all head and neck cancer cases; it mostly occurs in the oropharynx, hypopharynx, or oral cavity. The incidence of HNSCC has been increasing, and its prognosis is poor [1]. For example, in laryngeal cancer, except for glottic cancer, the early symptoms of supraglottic and subglottic types are usually more insidious and over 50% of patients are in the middle and advanced disease stages when they consult a doctor [2]. The comprehensive treatment of HNSCC includes surgery, radiotherapy, and chemotherapy [3]. Although the treatment of HNSCC has made great progress in recent years, early lymph node metastasis, aggressive growth, and other malignant pathological characteristics are important factors for its high postoperative recurrence and metastasis rate, low survival rate, and poor long-term efficacy [4]. Patients diagnosed with HNSCC have about a 50% chance of surviving the disease over the period of 5 years [5], and a stable and effective biological index is urgently required to guide treatment and predict prognosis.

Tumour aggressiveness and treatment resistance are affected by the interaction between tumour cells and their microenvironment. Infiltration exists in the tumour microenvironment [6]. The local tumour microenvironment is a unique and complex environment of tumour-host interaction that occurs during tumour progression. It is mainly composed of tumour cells and tumour-infiltrating lymphocytes (TILs), which can inhibit or promote tumour growth [7, 8]. TILs play a vital role in tumour formation, growth, invasion, and metastasis and have predictive prognostic value [9, 10]. In HNSCC, high levels of TILs often indicate a better prognosis and can be considered independent predictors of recurrence in patients with HNSCC [11–13]. TILs include T lymphocytes, macrophages, and dendritic cells. The relationship between these cells and the prognosis of patients with HNSCC and their influence mechanisms are not completely understood.

CD8+ T lymphocytes, also known as cytotoxic T lymphocytes, are the main components of T lymphocytes. Owing to the presence of major histocompatibility complex (MHC) class I molecules and lysis of tumour cells with perforin and granzyme, CD8^+^ T lymphocytes are the most powerful antitumour cells in the human body. Some scholars believe that the level of TILs can be considered an effective indicator for predicting prognosis. Among patients with HNSCC in whom CD8^+^ T lymphocyte infiltration is high; the level of TILs also exhibits reliable prognostic value [14–16]. Chen et al. [14] found that the 3-year overall survival (OS) (63.8%) of patients with a low ratio of CD8+ T/FOXP3^+^ T lymphocytes was considerably decreased in contrast with that of patients having a higher ratio. Furthermore, a lower ratio signifies that the balance shifts from the tumoricidal effects of CD8^+^ T lymphocytes to the immunosuppression of FOXP3^+^ T lymphocytes. Therefore, a balanced CD8^+^ T/FOXP3^+^ T lymphocyte ratio is an effective prognostic indicator for patients with HNSCC. Shimizu et al. [15] demonstrated that high levels of CD8^+^ T lymphocytes at tumour margins were significantly associated with a better prognosis. However, the clinical significance and prognostic value of CD8^+^ T cell-associated regulatory genes in HNSCC remain unclear, and further research is required.

In this study, we first screened CD8^+^ T cell-related genes through the immune cell datasets. A molecular subtype of HNSCC premised on CD8^+^ T cell-related genes was further developed, and its relationship with patient prognosis and clinical characteristics was evaluated. Eventually, the 8-gene signature was generated premised on CD8^+^ T cell-associated genes, which exhibited stable predictive power in evaluating the prognosis and immunotherapy response of patients with HNSCC.

## 2. Materials and Methods

### 2.1. Downloading and Prerocessing Data

A total of 13 datasets related to immune cell lines, including GSE8059, GSE6863, GSE59237, GSE49910, GSE42058, GSE39889, GSE37750, GSE28726, GSE28490, GSE27838, GSE27291, GSE23371, and GSE13906, were downloaded from the Gene Expression Omnibus (GEO) database. These datasets included the chip expression data of 14 distinct immune cells, which included natural killer T cells, CD4 T cells, plasmacytoid dendritic cells, dendritic cells, natural killer cells, gamma-delta T cells, monocytes, lymphocytes, immature dendritic cells, eosinophils, myeloid dendritic cells, CD8 T cells, neutrophils, and B cells (Supplementary Table 1).

For the processing of the immune cell data, the following processes were carried out:

1. The robust multiarray average (RMA) function of Affy was employed to process each immune cell dataset. The batch effect that was present between the different datasets was removed utilizing removeBatchEffect function of the limma package. Finally, the probe was transformed to a Symbol format according to the annotation file.

We used The Cancer Genome Atlas (TCGA) Genomic Data Commons (GDC) Application Programming Interface (API) to retrieve the TCGA–HNSCC RNA-seq data as well as clinical survival and characteristic information. The TCGA–HNSCC RNA-seq data were processed using the procedures that are listed as follows: (1) We eliminated the samples without clinical follow-up data. (2) We eliminated any samples that did not have survival time. (3) The samples that had no status were eliminated. (4) The Ensemble format was transformed to the Gene Symbol format. (5) The middle value was used for multiple expressions of Gene Symbols. (6) Genes that exhibited low expression (less than 1 and constituted more than 50% of the sample) were filtered out.

We downloaded the GSE65858 head and neck cancer dataset with survival time from the GEO database and processed it in the following steps: (1) we eliminated samples of normal tissue; (2) we eliminated the samples containing no clinical follow-up data; (3) we excluded any samples that lacked data on the OS rate; (4) the samples that had no status were eliminated; and (5) in accordance with the annotation file, the probe was transformed to the Symbol format.

After the two datasets were preprocessed, 499 samples were obtained from TCGA, and 270 samples were obtained from the GSE65858 dataset.  Table 1 depicts the clinical statistics of the samples collected.

### 2.2. Analysis of CD8 T Cell-Related Genes Premised on WGCNA

We examined the co-expressed coding genes and co-expression modules depending on the expression patterns of these genes utilizing the weighted gene co-expression network analysis (WGCNA). We then performed cluster analysis on the samples utilizing hierarchical clustering. The analysis was based on 179 expression patterns that were collected from the immune cell datasets. In addition to this, the Pearson correlation coefficient was utilised to determine the distance between each gene. The weighted co-expression network applied for the screening of co-expression modules was established with the help of the R software platform WGCNA. We demonstrated that the co-expression network conformed to the scale-free network, in which the logarithm of a node's connection degree of *k*, called log (*k*), was inversely linked to the logarithm of the node's appearance probability, called log (*P* (*k*)), and the correlation coefficient was >0.85. After that, the expression matrix was transformed to be an adjacency matrix, and thereafter, this adjacency matrix was modified to become a topological matrix. Clustering of genes was achieved utilizing the average-linkage hierarchical clustering technique premised on TOM. For each gene network, the least number of genes was adjusted to 150 as per the guidelines of the standard hybrid dynamic shearing tree. Following the completion of the dynamic shearing method for determining gene modules, we quantified each module' eigengenes before conducting cluster analysis on these modules. We created a new module with a height of 0.25, a deepSplit of 2, and a minModuleSize of 100 by merging the modules that were located near one another. Additionally, we adopted the R software function clusterProfiler (version 3.14.0) to analyse CD8 T cell-related genes by Kyoto Encyclopedia of Genes and Genomes (KEGG) pathway analysis and Gene Ontology (GO) functional enrichment analysis.

### 2.3. Construction of Molecular Typing on the Basis of CD8 T Cell-Related Genes

Clustering was performed on 499 HNSCC specimens that were part of the TCGA cohort utilizing ConsensusClusterPlus, and the cumulative distribution function (CDF) was generated to ascertain the optimum number of clusters. After observing the CDF delta area curve, relatively stable clustering results were selected. Furthermore, the immune subtype characteristics of various clusters were analysed. In addition, we used the same method to analyse the GSE cohort for validating the graft properties of molecular subtypes in different research cohorts.

### 2.4. Chemokines and Immune Checkpoint Genes Expression in CD8 T Cell Typing

Chemokines perform an integral function in the tumour onset and advancement. They can mediate various immune cells in the tumour microenvironment (TME) and help T cells to enter the tumour and subsequently affect tumour immunity and therapeutic effects. In this research, we analysed differences in the expression distribution of chemokines across the 2 subtypes. In the TCGA cohort, we computed the variations (differences) in gene expression between each of these genes. Additionally, we computed and contrasted the levels of expression of chemokine receptor genes in the various immune subtypes.

### 2.5. Development of a Prognostic Risk Model Premised on CD8 T Cell-Related Genes

#### 2.5.1. Acquisition of Training and Validation Sets

The TCGA dataset had 499 specimens, which were split at random into the training and the validation set. All of the data were first subjected to a random grouping, and then that grouping was repeated one hundred times with replacement so that the impact of random allocation bias on the reliability of subsequent modelling could be reduced. The ratio of 7 : 3 between the training set and the validation set served as the basis for the implementation of group sampling. The following criteria were utilised to choose the training and validation sets that were found to be the most appropriate: (1) Both groups had a comparable age distribution, patient death ratio, and time spent on follow-up; (2) Following the clustering of the gene expression patterns of the two randomized group datasets, the number of binary classification samples remained relatively unchanged. A total of 349 and 150 samples were eventually obtained in the training and test datasets, respectively.  Table 2 displays some of the information that was gathered from the training and validation sets in the TCGA dataset. The chi-square test was performed on both the training and test samples. Our categorization of the data was validated by the findings, which showed that there were no significant differences between the two groups (*P* > 0.05).

### 2.6. Development of a Model Leveraging LASSO Regression

To execute a univariate Cox proportional hazard regression analysis for T cell-associated genes and survival data from the training set, the survival coxph program from the R software was utilised. We identified the potential prognostic genes by setting the cut-off value at *P* < 0.05. To minimize the overall number of genes included in the risk model, we implemented LASSO regression and additionally compressed the prognostic genes. Compression estimation was achieved with the use of the LASSO model. Constructing a penalty function that can concurrently compress certain coefficients and set others to zero led to the creation of a more sophisticated model than would have otherwise been possible. As a consequence, the benefit of subset shrinking was retained, even though it resulted in a skewed estimate when applied to the processing of data containing multicollinearity. It has the potential to realise the variable selection while simultaneously assessing parameters and can better cope with the multicollinearity issue that arises in regression analysis.

## 3. Results

### 3.1. Determination of the CD8 T Lymphocyte Marker Genes

We merged 13 immune cell datasets, eliminated the batch effect, and subsequently removed the influence before and after the batch effect through PCA analysis (Supplementary Figures 1(a) and 1(b)). The results revealed that the samples of different datasets were scattered before eliminating the batch effect and were mixed after eliminating the batch effect.

### 3.2. Analysis of CD8 T Cell-Related Genes Premised on WGCNA

**Figure 1:**
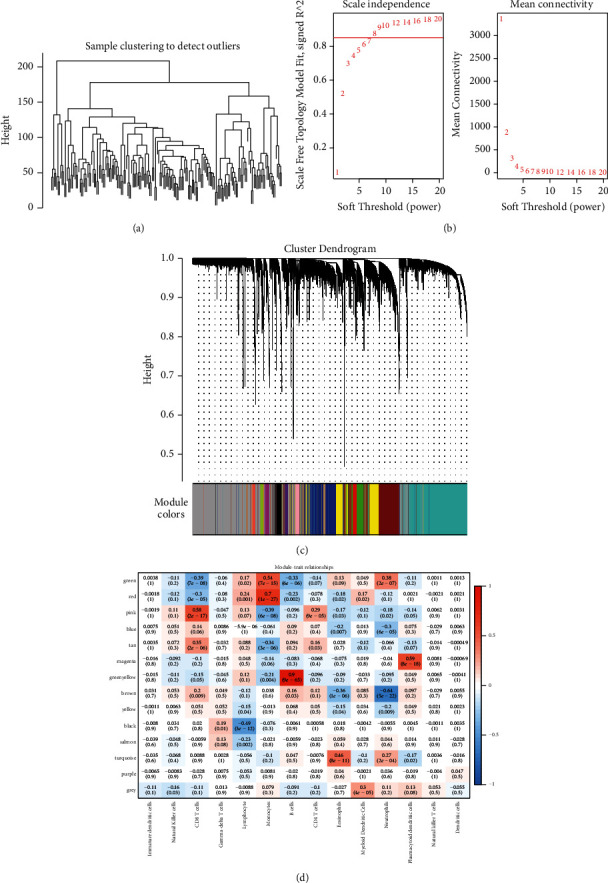
(a) A representative cluster analysis sample. (b) An examination of the topology of the network for a series of different soft threshold powers. (c) The gene dendrogram and the colours of the modules. (d) Analysis of the relationship between 14 different modules and each clinical phenotype.

The hierarchical cluster analysis of expression profiles of 179 immune cell datasets is shown in Figure 1(a). The R software program WGCNA was utilised to create a weighted co-expression network, and 8 was selected as the soft threshold. To guarantee that the network would be scale free, we decided to make *β* equal to 8 (Figure 1(b)). Following the completion of the dynamic shear approach for discovering gene modules, we then computed each module's eigengenes. We subsequently subjected the modules to cluster analysis and combined the modules located in close proximity to each other into a new module with a height of 0.25, deepSplit of 2, and minModuleSize of 150. We ended up obtaining 14 modules (Figure 1(c)). The grey module included a group of genes that cannot be incorporated into any of the other modules. We subsequently evaluated the link between each module and immune cells as shown in Figure 1(d). It was evident that the pink module was the most significantly positively linked to CD8 T cells; however, it exhibited less correlation with other immune cells. Furthermore, it included a total of 446 genes.

### 3.3. Analysis of the Functional Enrichment of CD8 T Cell-Related Genes

We further adapted the R software function clusterProfiler (version 3.14.0) to perform GO functional enrichment and KEGG pathway analyses on CD8 T cell-related genes. For the GO functional annotations of genes, 284 items were annotated to biological process (BP) with significant differences (*P* < 0.05), and the annotation results of the first 10 items are shown in Supplementary Figure 2(a). An aggregate of 26 items was annotated to molecular function (MF) with significant differences (*P* < 0.05), and the annotation results are shown in Supplementary Figure 2(b). In total, 46 items were annotated to cellular component (CC) with significant differences (*P* < 0.05), and the annotation results of the first 10 items are shown in Supplementary Figure 2(b).

Premised on the analysis of KEGG pathway enrichment of the marker gene, 33 pathways were annotated to be significant (*P* < 0.05). The results of the first 10 annotations are shown in Supplementary Figure 2(d). The findings of the gene annotation indicated that these genes are strongly linked to immune pathways and functions.

### 3.4. Molecular Typing Premised on CD8 T Cell-Related Genes

#### 3.4.1. Construction of Molecular Typing Depending on CD8 T Cell-Related Genes

**Figure 2:**
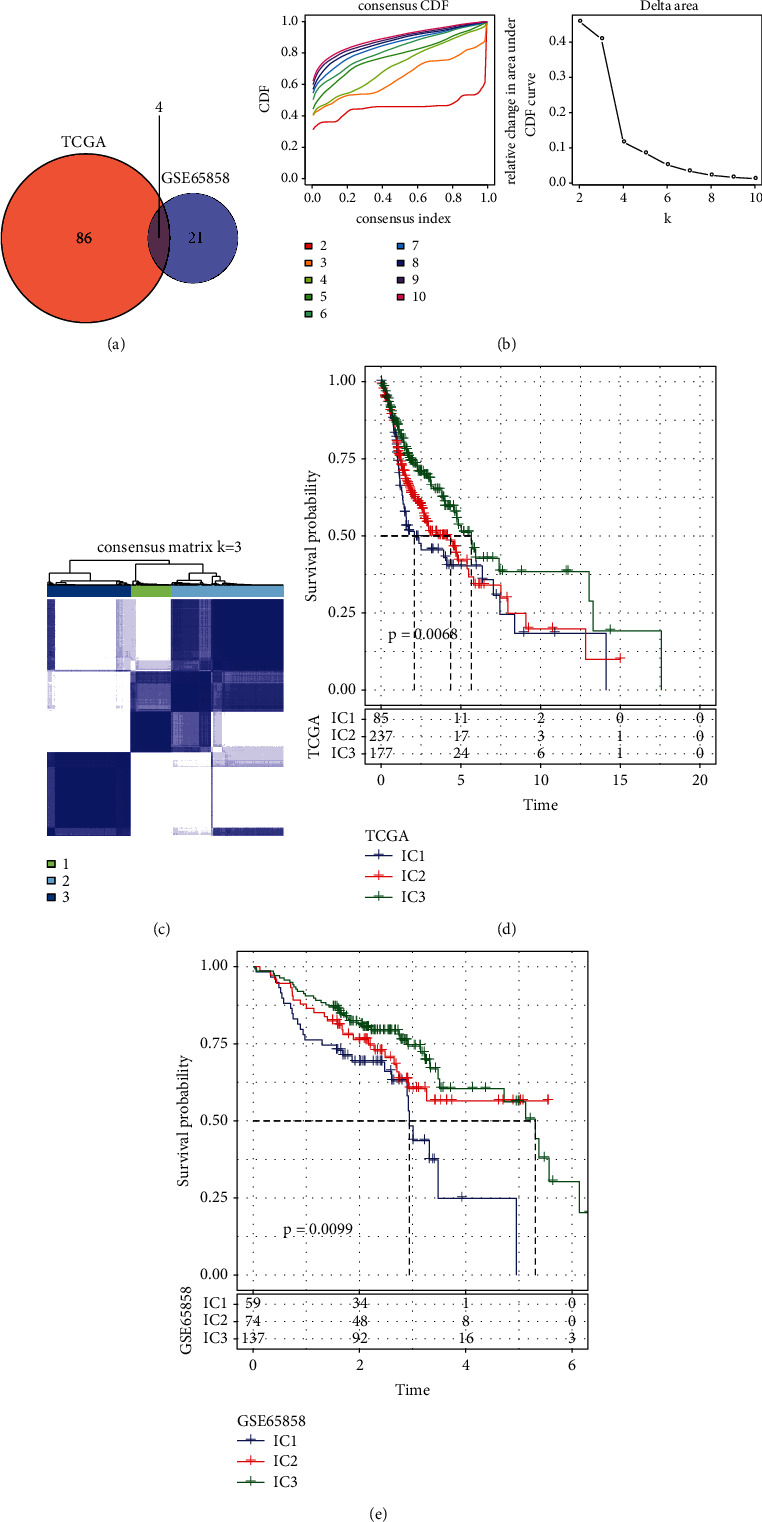
Immune cluster in HNSCC. (a) A Venn diagram showing the intersection of CD8 T cell-related genes that have a substantial prognostic link in the two cohorts. (b) The CDF curve of the samples from the TCGA cohort, as well as the CDF delta area curve of consensus clustering, which displays the relative fluctuation in the area under the CDF curve from category number *k* to category number k-1 for each category. The category number k is shown by the horizontal axis, whereas the relative fluctuation in the area under the CDF curve is represented by the vertical axis. (c) Heat map of sample clusters taken at a consensus *k* = 3. (d) Kaplan–Meier curve of the prognostic relationship among the three subtypes in the TCGA dataset. (e) Kaplan–Meier curve of the prognostic relationship among the three subtypes in the GSE65858 dataset.

The TCGA and GSE65858 gene datasets were used as the basis for our first univariate analysis of genes associated with CD8 T cells. The univariate survival analysis revealed that a total of 90 genes in the TCGA cohort and 25 genes in the GSE65858 cohort were related to prognosis (Supplementary Tables 2 and 3). Only four intersections were found between them, as shown in Figure 2(a), which indicated that the consistency of CD8 T cell-related genes among datasets from different platforms was poor, and a single CD8 T cell-related gene was quite different in different cohorts. Therefore, we further used 111 CD8 T cell-related genes associated with prognosis in the 2 datasets for further investigations (*P* < 0.05).

Within the TCGA cohort, the program ConsensusClusterPlus was utilised to cluster 499 HNSCC specimens. CDF was used to arrive at an answer for the optimum possible number of clusters. Based on the CDF delta area curve, it was evident that there was relatively stable clustering when the Cluster was selected as 3 (Figure 2(b)). We eventually selected *k* as 3 to obtain two CD8 T cell-related subtypes (immune cluster (IC)) (Figure 2(c)). As a result of our further research into the prognosis features of these three immunological subtypes, we discovered that there were considerable differences between their respective prognostic profiles, as shown in Figure 2(d). IC1 exhibited a poor prognosis, whereas IC3 exhibited a good prognosis. In addition, we used the same method to observe the same phenomenon in the GSE cohort as shown in Figure 2(e). According to these findings, the three molecular subtypes that were premised on the genes associated with CD8 T cells were applicable in different research cohorts.

### 3.5. Comparison of Clinical Features between Immunotypes

In the TCGA dataset, we did a comparison of the distribution of various clinical characteristics across the three distinct subtypes to observe whether there were any differences. The following findings were discovered as a consequence of these tests: (1) There was a substantial difference between the 3 subtypes in terms of their survival rates. The IC1 group with a dismal prognosis had a greater number of fatalities. (2) The proportion of *T* staging in the three subtypes was significantly different, and the percentage of T2, T3 and T4 were greater in the IC1 group. (3) The percentage of grades within the three groups varied considerably. (4) The percentage of smoking varied substantially among the 3 groups. (5) The percentage of HPV in the 3 groups varied considerably (Supplementary Figures 3(a)—3(f)).

### 3.6. Expression of Chemokines and Immune Checkpoint Genes in CD8 T Cell Typing

**Figure 3:**
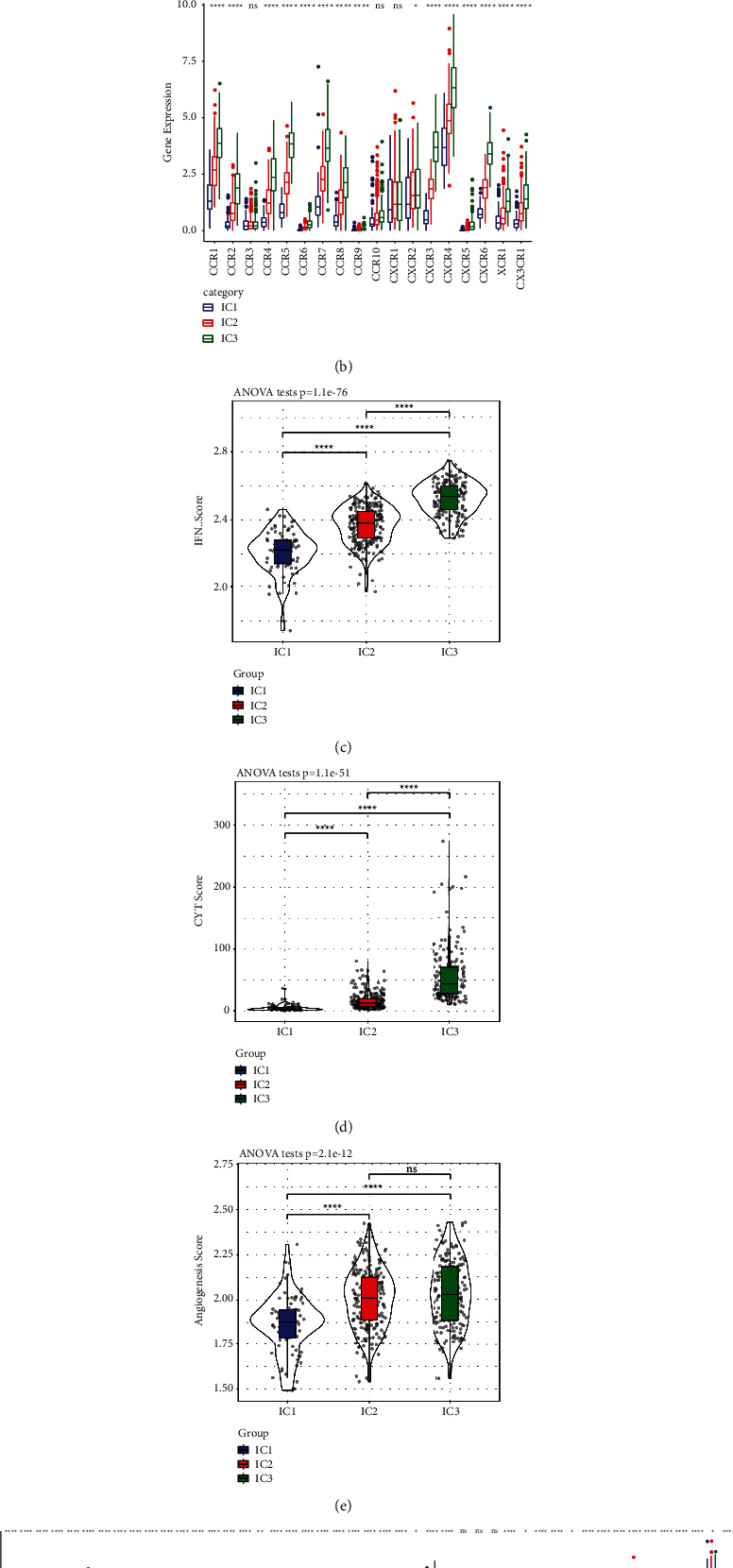
(a) Variations in the expression distribution of chemokines derived from the TCGA cohort. (b) Variations in the expression distribution of chemokine receptors derived from the TCGA cohort. (c) Variations in the IFN-*γ* score distributions among different subgroups in the TCGA cohort. (d) Variations in the lytic activity of immune T cells across the various subgroups. (e) Differences in angiogenesis scores among different subgroups. (f) Variations in immune checkpoint genes expression and distribution of obtained from the TCGA cohort, the significance of which was statistically tested using analysis of variance. ^∗^*P* < 0.05; ^∗∗^*P* < 0.01, ^∗∗∗^*P* < 0.001, and ^∗∗∗∗^*P* < 0.0001.

Studies have found that chemokines perform a fundamental function in tumour onset and progression. Chemokines can mediate various immune cells in the TME and help T cells to enter the tumour and affect tumour immunity as well as therapeutic effects. Therefore, we analysed the differential expression distribution of chemokines between the two groups. We examined the differences in genes within the TCGA cohort as shown in Figure 3(a). Of the 41 chemokines, 33 (80.5%) exhibited substantial differences between groups, which illustrated that the degree of immune cell infiltration among subtypes was different. Differences were also observed in tumour progression and immunotherapy effects. Furthermore, we calculated and contrasted the chemokine receptor gene expression in immune subtypes as shown in Figure 3(b) and found that 15 (83.33%) of the 18 chemokine receptor genes exhibited considerable differences in the expression of immune subtypes.

CD8+ T cells in the TME may secrete interferon-gamma (IFN-*γ*), which can upmodulate IDO1 and PD-1/PD-L1 gene expression [17, 18]. Studies have demonstrated that the up modulation of IDO1 expression is favourably linked to dismal prognosis, tumour progression as well as metastasis [19, 20]. We extracted Th1/IFN-*γ* gene signatures from a previous study [21] and computed each patient's IFN-*γ* score with the aid of the single-sample Gene Set Enrichment Analysis (ssGSEA) method. Significant differences were found in the IFN-*γ* scores across the 3 subtypes, and the IC3 subgroup had a higher IFN-*γ* score, whereas the IC1 subgroup exhibited the least IFN-*γ* score as shown in Figure 3(c).

In a study by Rooney [22], the average value of PRF1 and GZMA expression was used to evaluate the intratumoral immune T cell lytic function of each patient. Significant differences were found among the three subgroups as shown in Figure 3(d). IC3 had the highest immune T cell lytic activity, whereas IC1 had the lowest activity.

The angiogenesis-related gene set was retrieved from an earlier study [23], and the angiogenesis score of each patient was evaluated. Significant differences were observed among different subgroups as shown in Figure 3(e); the angiogenesis scores of IC2 and IC3 were significantly higher than that of IC1.

Furthermore, we acquired 47 immune checkpoint-associated genes from an earlier research report [21] and analysed the differences among these genes in distinct immune subtypes. The findings indicated that 44 (93.62%) of these genes exhibited significant differences as shown in Figure 3(f). These findings illustrated that there were variations in immunotherapy response across the subgroups. Most immune checkpoint-associated genes were expressed at a high level in IC3, including the genes LAG3, CTLA4, PDCD1, PDCD1LG2, and IDO1.

### 3.7. Immune Properties and Pathway Features of Various Immunotypes

**Figure 4:**
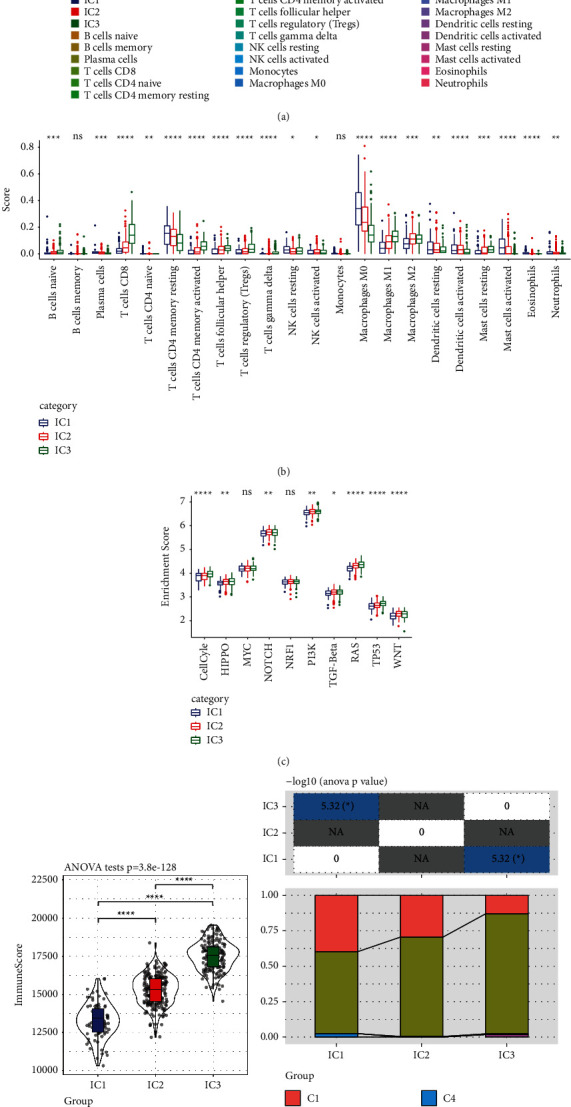
(a) The ratio of 22 distinct immune cell components distributed among a variety of subgroups. (b) Variations across the various subgroups with regard to 22 distinct immune cell constituents of the samples. (c) Variations in the scores assigned to ten pathways linked to abnormalities seen in tumours among the various subgroups. (d) Scores on immune infiltration that varied significantly among the various subgroups. (e) Molecular subtypes were compared with six other pan-cancer immune molecular subtypes that had previously been described.

The CIBERSORT technique was adopted to determine the scores of 22 distinct immune cells present in each sample included in the TCGA dataset.  Figure 4(a) illustrates the distribution patterns of these immune cell scores among the four distinct subgroups. Differences between the subtypes of immune cells are shown in Figure 4(b). We observed that there were substantial variations in immunological features across the various subgroups, which we determined by comparing the immune cell scores of each subgroup. Significantly high differences were found in subtypes such as CD8 T cells; resting memory CD4 T cells and macrophages M0, M1, and M2; these subtypes might perform an integral function in HNSCC.

Based on the variations observed between the two subgroups in the previous 10 oncogenic pathways [24], it was evident that 8 of these pathways exhibited significant differences among the subtypes (Figure 4(c)).

Immune infiltration analysis illustrated that IC3 exhibited the highest immune microenvironment infiltration score, and IC1 had the lowest score as shown in Figure 4(d). As per the findings from the differential expression analysis of immune checkpoints in distinct subtypes, the expression level of most immune checkpoint-related genes was considerably elevated in IC3 as opposed to that in IC1, which may be attributed to the better prognosis observed in IC3.

To evaluate the connection between our molecular subtypes and six previously reported pan-cancer immunotypes, we acquired the molecular subtype-related data of these samples from earlier studies [25] for comparison (the ratio of C1 and C2 subtypes was 98.58%, and the prognosis of the C2 subgroup was more favourable in contrast with that of the C1 subgroup). Substantial variations were discovered in the immunophenotyping of the previously reported pan-cancer immunotypes as shown in Figure 4(e).

The C1 subtype exhibiting a poor prognosis constituted a remarkably elevated proportion of the IC1 subtype, which we had defined, than that of IC3, and the proportion of the C2 subtype with a slightly better prognosis was considerably elevated in IC3 as opposed to that in IC1, which was consistent with our definition that IC1 had an unfavourable prognosis. This analysis illustrated that the three subtypes we defined could be used as supplements to the six subtypes reported in the previous study.

### 3.8. Differential Analysis of Subtypes Using TIDE

**Figure 5:**
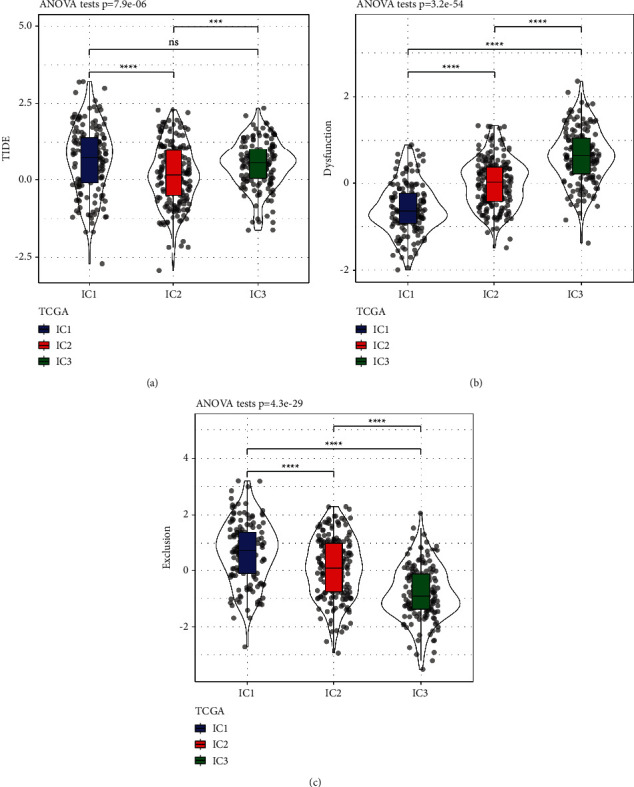
(a) There are variations in TIDE score among the various subtypes of TCGA. (b) T cell dysfunction score variations across a variety of subtypes as measured from TCGA. (c) There are variations in T cell rejection scores across the various subtypes measured via TCGA.

We analysed the differences in chemotherapy and immunotherapy across distinct molecular subtypes. We used the TIDE software (https://tide.dfci.harvard.edu/) to examine the possible clinical impacts of immunotherapy in our defined molecular subtypes. When the TIDE prediction score was greater, it meant that there was a greater chance of immunological evasion; this implied that the patients had a less likelihood of benefiting from immunotherapy. As shown in Figure 5(a), we found that the TIDE scores of IC1 and IC3 were remarkably elevated in contrast with that of IC2 in the TCGA dataset, implying that IC1 and IC3 could benefit from immunotherapy more than IC2 can. Simultaneously, we contrasted the variation in the predicted T cell rejection and dysfunction scores across distinct molecular subtypes. The findings revealed that IC1 exhibited a lower T cell dysfunction score, as shown in Figures 5(b) and 5(c), and IC1 exhibited an elevated T cell rejection score, whereas IC3 exhibited a decreased T cell rejection score. This may be attributed to the grim prognosis of IC1 and the favourable prognosis of IC3.

### 3.9. Analysis of Variations in Chemotherapy/Immunotherapy among Immune Subgroups

**Figure 6:**
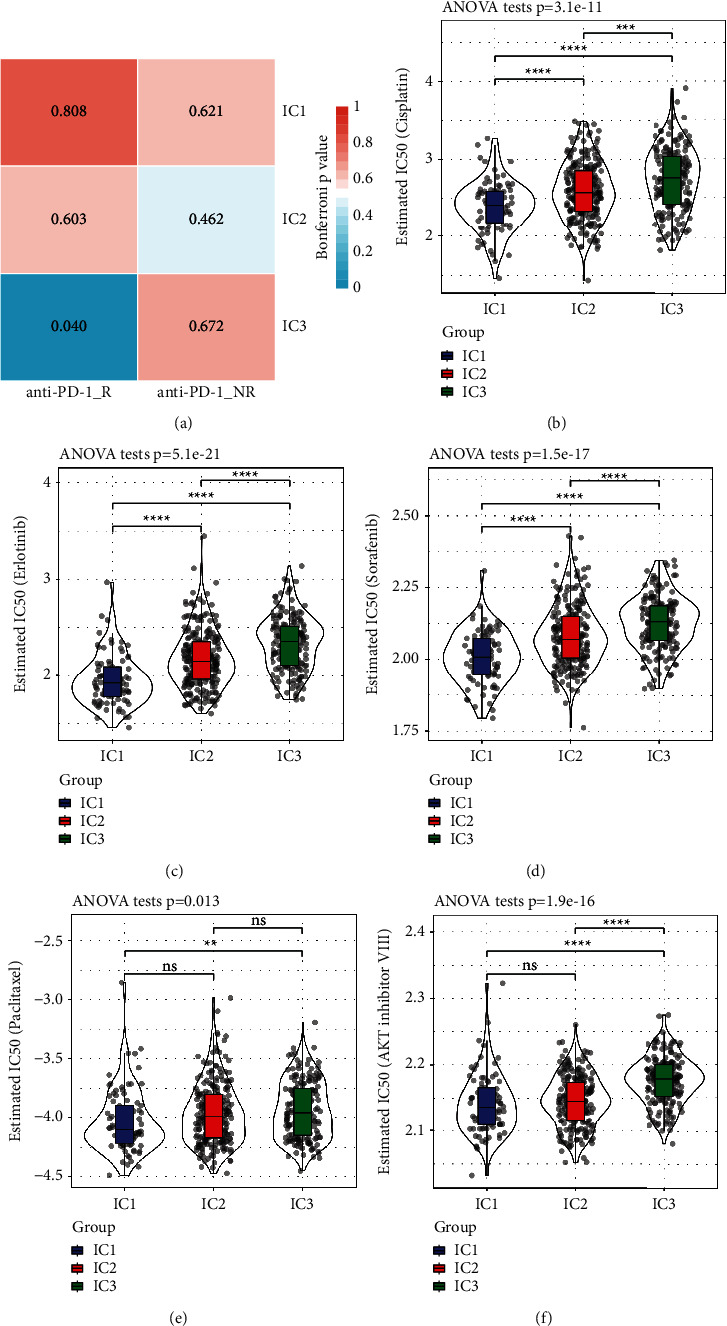
(a) TCGA subclass mapping manifested that IC3 was highly responsive to anti-PD-1 (Bonferroni-corrected *P* < 0.05). (b–f) Box plots displaying the predicted IC50 values for each specimen included in the TCGA dataset.

We examined the differences in the response of patients belonging to various immune molecular subtypes to chemotherapy and immunotherapy. We used subclass mapping to identify similarities among the subtypes we defined and patients under immunotherapeutic treatment in the GSE78220 dataset. A greater degree of similarity exists when the *P*-value is smaller. As a consequence of this, we discovered that the IC3 subtype, which was studied using the TCGA dataset, was more responsive to anti-PD-1. Simultaneously, we also analysed the response of distinct subtypes to standard chemotherapeutic drugs, such as cisplatin, erlotinib, sorafenib, paclitaxel, and AKT inhibitor VIII, and found that contrasted to subtypes, the IC1 was more sensitive to these five drugs (Figures 6(a)–6(f)).

### 3.10. Establishment of a Prognostic Risk Model Premised on CD8 T Cell-Related Genes

#### 3.10.1. Randomization of Training Set Sample Groupings

The TCGA dataset contained a sum of 499 samples, which were split at random into training and validation sets. All of the data were grouped at random a hundred times with replacement in the early stages of the modelling process to avoid variations in the random allotment from affecting the consistency of the final models. The ratio of 7 : 3 in the training set to the verification set was used as the basis for group sampling. The following criteria were adopted to choose the training and validation sets that were found to be the most appropriate: (1) Both groups had a comparable age distribution, patient death ratio, and time spent on follow-up; (2) Once the gene expression patterns of the two randomized datasets were clustered, the number of binary classification samples that were present remained relatively unchanged. Eventually, in the training set, 349 samples were collected, whereas the test set contained 150 samples.  Table 2 presents the information gathered from the TCGA dataset's training and validation sets. The chi-square test was performed on the samples derived from both the training set and the test set. According to the findings, our method of classification was appropriate, and there were no discernible variations across the subgroups (*P* > 0.05).

### 3.11. Single-Factor Risk Assessment of the Training Set

R's survival coxph program was employed to carry out univariate Cox proportional hazard regression for CD8 T cell-associated genes and survival data in the training set. The cut-off value used for filtering was determined to be *P* < 0.05. Eventually, 68 genes were found with significant variations. The results of the univariate Cox analysis are shown in Supplementary Table 4.

### 3.12. Multifactorial Risk Analysis of the Training Set

**Figure 7:**
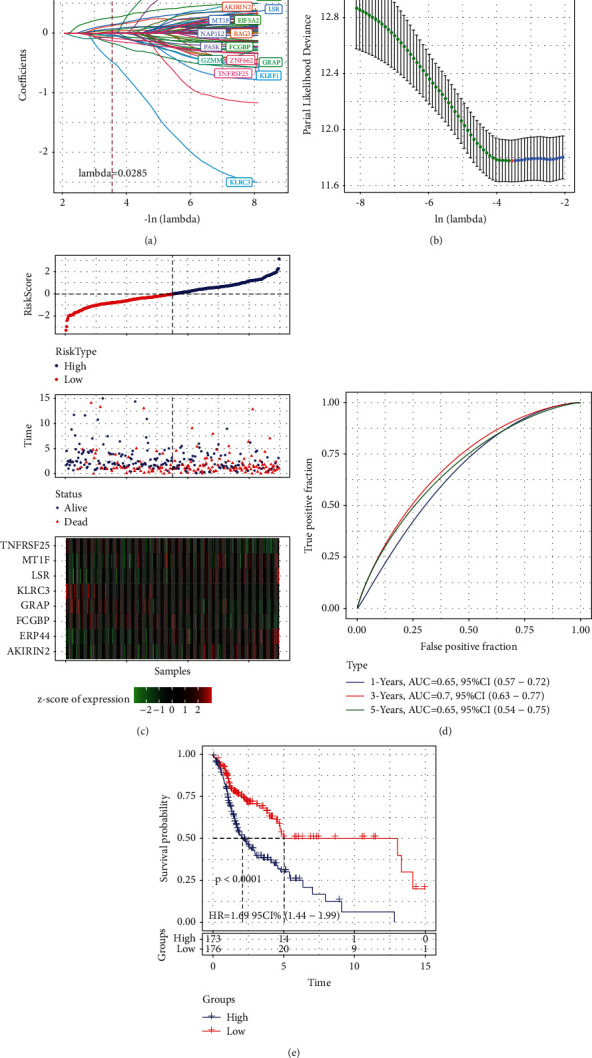
(a) The trajectories of change for each independent variable; The log value of the independent variable lambda is represented on the horizontal axis, whereas the coefficient of the independent variable is depicted on the vertical axis. (b) Illustration of each lambda's confidence interval. (c) Eight genes' expression levels, survival time, risk score, and survival conditions in the TCGA training set. (d) AUC values of ROC curves of the eight-gene signature. (e) Distribution of the KM survival curve for the eight-gene signature in the training set.

The TCGA and GSE datasets together yielded a sum of 68 CD8 T cell-related genes that were associated with the prognosis. However, because of the high quantity, these genes did not facilitate clinical detection. We additionally compressed these 68 genes by using lasso regression to minimize the total number of genes employed in the risk model. To conduct the LASSO Cox regression analysis, we used the glmnet software package in R.  Figure 7(a) presents the results of an analysis conducted on the changing trajectory of each independent variable. It was noticed that the proportion of independent variable coefficients that tended toward zero progressively grew with a steady rise in the value of lambda. We used ten-fold cross-validation to create the model and analysed the confidence interval (CI) under each lambda as shown in Figure 7(b). It was evident from the figure that the model attained an optimum state at a lambda value of 0.0285. Hence, we selected 15 target genes when lambda was 0.0285 in the next step.

The Akaike information criterion (AIC) was utilised in stepwise regression, which took into account the statistical appropriateness of the model as well as the number of parameters that were applied to fit the model. The stepAIC approach that was included in the MASS package commenced with the most sophisticated model and then removed a variable in order to lower the AIC value. The smaller the value, the better the model, which implied that the model used lessor parameters to attain a satisfactory degree of fit. Eventually, with the help of this algorithm, we were able to cut the number of genes down from 15 to 8. These genes were ERP44, AKIRIN2, GRAP, KLRC3, FCGBP, LSR, TNFRSF25, and MT1F. The following was the equation for the signature incorporating 8 genes: RiskScore = 0.441 ^*∗*^ ERP44 + 0.283 ^*∗*^ AKIRIN2-0.400 ^*∗*^ GRAP − 1.038 ^*∗*^ KLRC3 − 0.082 ^*∗*^ FCGBP + 0.224 ^*∗*^ LSR − 0.265 ^*∗*^ TNFRSF25 + 0.138 ^*∗*^ MT1F.

### 3.13. Development and Assessment of Risk Models

We determined each sample's risk score by contrasting it to the expression level of the samples in the TCGA training dataset, after which we examined how the risk scores were distributed across the samples, as shown in Figure 7(c). In addition, we examined the ROC of risk scores for prognostic classification with the aid of the timeROC software tool in the R programming. We examined the effectiveness of the prognostic categorization over 1, 3, and 5 years. As shown in Figure 7(d), the value of the area under the curve (AUC) in the model was high. Lastly, we applied zscore to RiskScore and subdivided the samples whose risk score was >0 into high-risk groups and those whose risk score was <0 into low-risk groups. A Kaplan–Meier (KM) curve was also constructed. As shown in Figure 7(e), both the groups exhibited a remarkable difference with *P* < 0.0001.

### 3.14. Validation of the Risk Model

We computed each sample's risk score depending on the expression level of the samples in the TCGA validation dataset and analysed the risk score distribution of the samples as shown in Supplementary Figure 4(a). Additionally, we examined the ROC values of risk scores for prognostic classification by employing the timeROC software tool in the R programming language. We evaluated the accuracy of the prognostic prediction over 1, 3, and 5 years. As shown in Supplementary Figure 4(b), the model had a high AUC value. Eventually, we applied zscore to Riskscore and classified the samples whose risk score was >0 into high-risk groups and those whose risk score was <0 into low-risk groups. A KM curve was also constructed. As shown in Supplementary Figure 4(c), both the groups exhibited a significant variation with *P* < 0.0001.

We determined each sample's risk score by comparing the expression levels of the samples across all of the TCGA datasets, and thereafter, we analysed how the risk scores were distributed, as shown in Supplementary Figure 5(a). Moreover, we assessed the ROC of risk scores for prognostic classification with the aid of the timeROC program that is included in R. We evaluated its prognostic accuracy over 1, 3, and 5 years. As shown in Supplementary Figure 5(b), the model exhibited an elevated AUC value. Eventually, we applied zscore to Riskscore and classified the samples whose risk score was larger than 0 into high-risk groups and those whose risk score was <0 into low-risk groups. A KM curve was also constructed. As shown in Supplementary Figure 5(c), both the groups exhibited a significant variation with *P* < 0.0001.

Supplementary Figure 6(a) displays the distribution of the risk score of the independent verification dataset GSE65858. In addition, we carried out the ROC analysis of the prognostic categorization of risk scores by using the timeROC package as shown in Supplementary Figure 6(b), we analysed to determine the prognostic predictive accuracy over 1, 3, and 5 years. Eventually, we applied scores to Riskscore and subdivided the samples whose risk score was larger than 0 into high-risk groups and those whose risk score was smaller than 0 into low-risk groups. A KM curve was also constructed. As shown in Supplementary Figure 6(c), both the groups exhibited a significant variation with *P* < 0.0001.

### 3.15. The Performance of Risk Scores in Various Clinical Parameters and Molecular Subtypes

**Figure 8:**
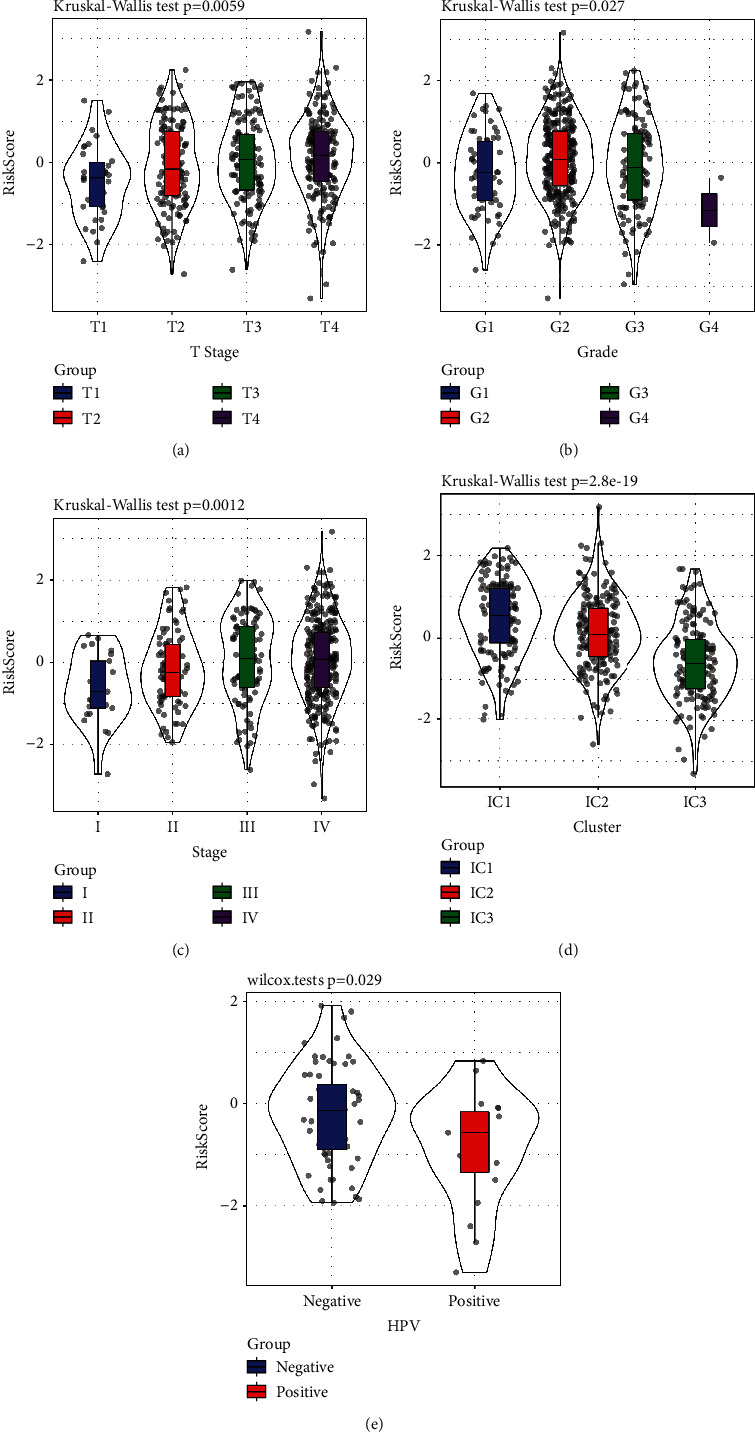
Comparison of the distribution of the risk scores of the CGA dataset across clinical feature groups.

We examined the distribution of risk scores from the TCGA dataset across the different clinical characteristic groups, and we deduced that remarkable variations were found in *T* stage, grade, subtype grouping, and HPV (Figures 8(a)–8(e); *P* < 0.05). The IC1 group that exhibited the most unsatisfactory prognosis also had the greatest risk score, whereas the IC1 group had the most favourable prognosis and the least risk score.

### 3.16. Relationship of Risk Scores with Channels

**Figure 9:**
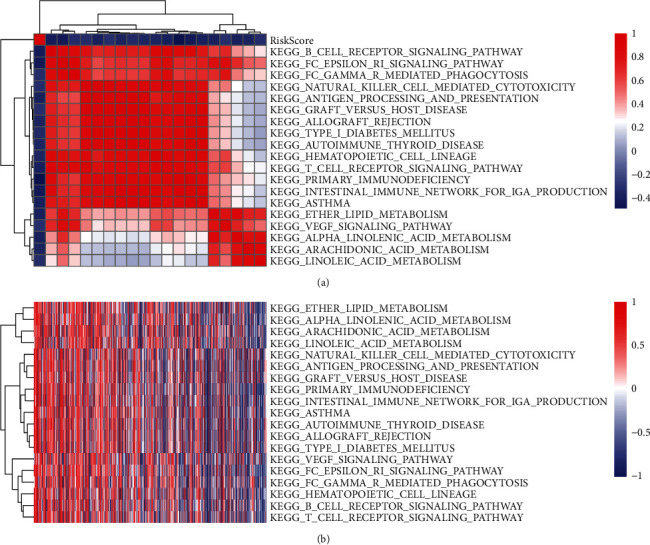
(a) Correlation coefficients between KEGG pathways and risk scores larger than 0.3 are clustered. (b) The KEGG pathway that had a risk score above 0.3 exhibited a distinct ssGSEA score, and this score changed as the risk level grew. The samples are shown along the horizontal axis, and the increasing risk scores can be observed moving from left to right.

The ssGSEA analysis was done by selecting the gene expression patterns that correspond to the various samples and we employed the R software program GSVA to perform the analysis. This allowed us to examine the link between the risk scores of the various samples and the biological functions. After calculating the scores that each sample generated on the various functions, we determined the ssGSEA score that corresponded to each function for each sample. Further analysis was done to ascertain the association that exists between these functions and risk scores. Features with a correlation larger than 0.3 were chosen. As shown in Figure 9(a), we found that 19 of them were inversely linked to the sample risk scores. In total, 19 KEGG pathways were chosen as the most relevant. An analysis of clustering was carried out premised on the enrichment scores of the groups, as shown in Figure 9(b). These 19 pathways, including KEGG_ETHER_LIPID_METABOLISM, KEGG_ARACHIDONIC_ACID_METABOLISM, KEGG_B_CELL_RECEPTOR_SIGNALING_PATHWAY, and KEGG_T_CELL_RECEPTOR_SIGNALING_PATHWAY, were related to immune function and metabolism and decreased with an increase in risk scores.

### 3.17. Univariate and Multivariate Analyses of the 8-Gene Signature

**Figure 10:**
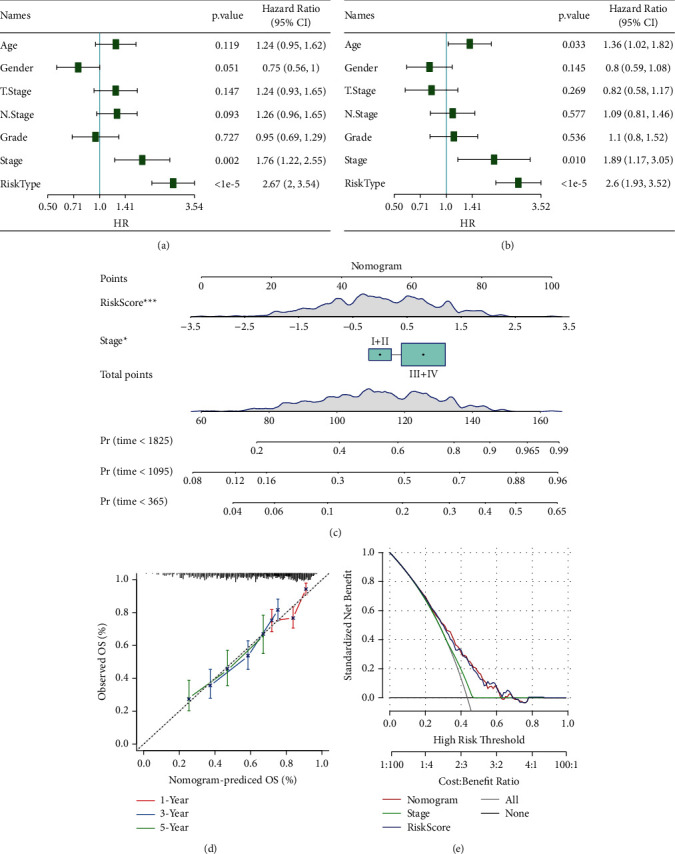
(a) Univariate analysis of all TCGA datasets. (b) Multivariate analysis of all TCGA datasets. (c) Construction of a multivariate nomogram. (d) Correction curve. (e) DCA chart.

To discover independent clinical applications of the 8-gene signature model, each variable was included for univariate and multivariate Cox analyses. The findings illustrated that risk scores were always substantially linked to survival (hazard ratio [HR] = 2.6, 95% CI = 1.93–3.52,*P* < 1e − 5) regardless of univariate or multivariate Cox analysis (Figures 10(a) and 10(b)). According to the findings, our 8-gene signature model displayed good prediction performance when used in clinical settings.

### 3.18. Construction of Nomograms and Forest Diagrams with Risk Scores and Clinical Parameters

The findings of a risk model can be displayed in a nomogram in a manner that is both more intuitive and effective. It is more convenient to predict the outcome using a nomogram. The length of a straight line in a nomogram is used to denote the extent to which distinct factors contributed to the outcome, and it also demonstrates how the contribution of distinct values to those variables affected the final outcomes. We built a nomogram model by combining stage and risk scores. We employed all TCGA datasets to build a nomogram for the combination of stage and risk scores (Figure 10(c)). The findings showed that risk scores had the largest influence on the rate of survival prediction, which suggested that the risk model incorporating eight genes could more accurately anticipate the outcome of the patient's condition. The correction curve demonstrated that the model exhibited adequate accuracy (Figure 10(d)). Furthermore, we made the DCA diagrams of the stage, risk score, and nomogram. According to the findings, the nomogram exhibited a higher degree of clinical applicability (Figure 10(e)).

### 3.19. Model for the Prediction of Risks Associated with Immunotherapy

**Figure 11:**
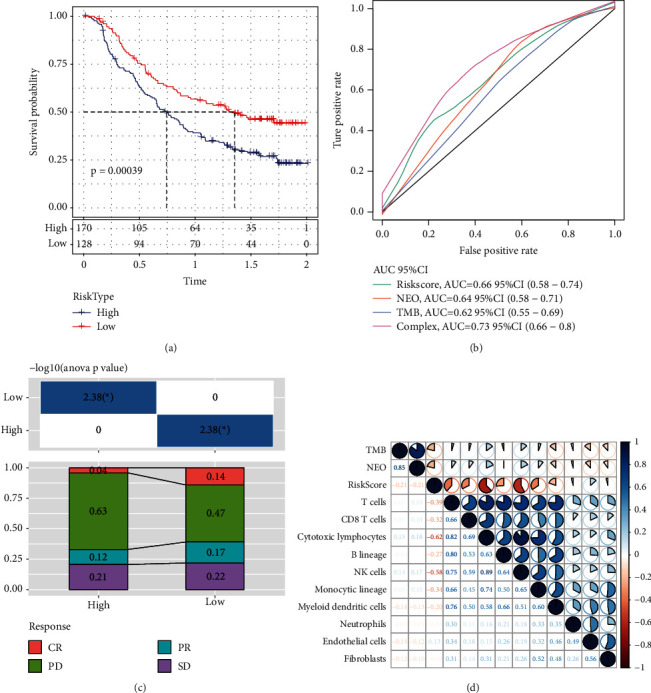
(a) Imvigor210 dataset' KM curve. (b) The Imvigor210 dataset's ROC curve. (c) Corresponding stacked graphs of immunotherapy among different groups of the Imvigor210 dataset. (d) Correlation between risk scores of the Imvigor210 dataset and immune scores, TMB and NEO.

There are not a lot of reliable prognostic indicators available for immunotherapy at the moment. The discovery of novel prognostic indicators is necessary for the further development of sophisticated immunotherapy. We discovered an immunotherapy dataset that had transcriptome data so that we might investigate whether the 8-gene model may accurately anticipate the advantages of immunotherapy. Imvigor210 collected expression data from human mUC specimens taken from patients responsive or unresponsive to anti-PD-L1 immunotherapeutic treatment. According to the KM curve, it was determined that patients with mUC undergoing immunotherapy with a higher risk score experienced a worse chance of survival (Figure 11(a)). The ROC curve demonstrated that the model combined with the risk score had a higher AUC value (Figure 11(b)). There were statistically significant variations discovered between the immunotherapy response and nonresponse scores in the high- and low-risk groups (Figure 11(c)). We utilised MCPcounter to calculate the immune cell score of Imvigor210 specimens and analyse the link between risk scores and TMB, NEO, and immune cell scores. The results revealed that risk scores exhibited an inverse link to NEO and TMB, and there was not much correlation with immune cell scores (Figure 11(d)).

**Figure 12:**
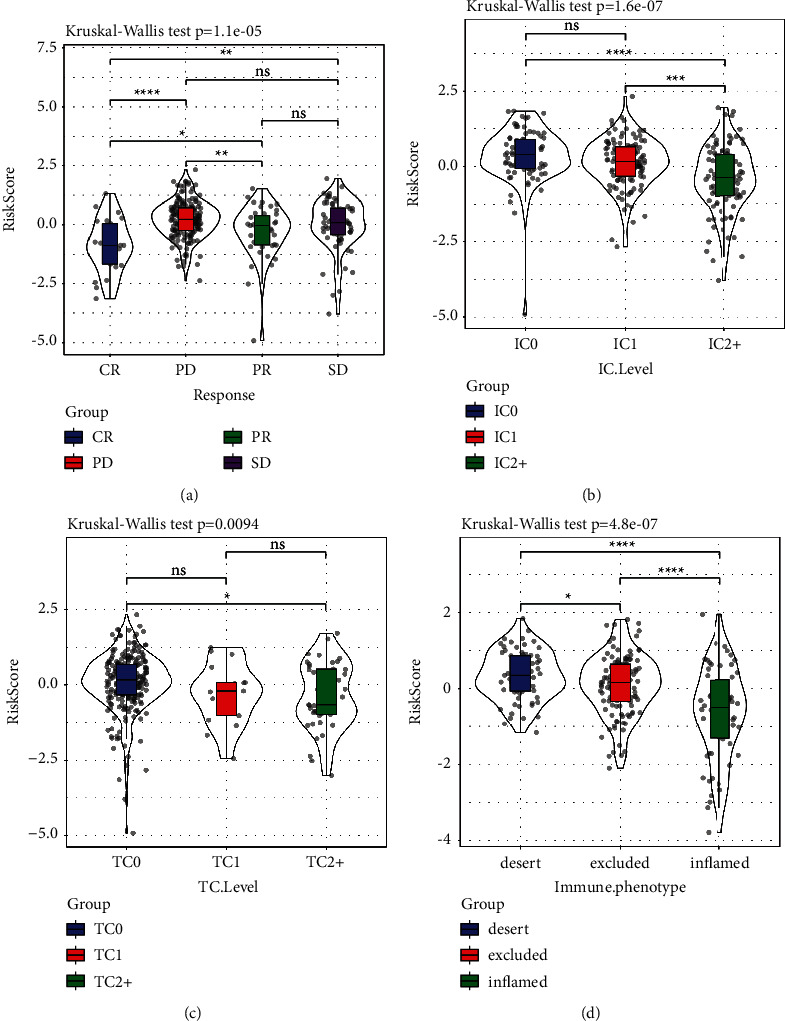
(a) Risk score differences among immunotherapy effectiveness groups. (b) Risk score differences among immune cell groups. (c) Risk score differences among tumour cell groups. (d) Difference in risk scores among immunophenotype groups (IP: Immune phenotype; TC: tumour cell; IC: immune cell).

We examined the variations in risk scores that existed between these groups. The results revealed significant variations in the effectiveness of risk scores and immunotherapy (Figure 12(a)). Significant differences were observed among the risk scores of immune cell groups (Figure 12(b)). Differences were found among risk scores of tumour cell groups (Figure 12(c)). Significant differences were found among the risk scores of immunophenotype groups (Figure 12(d)).

## 4. Discussion

In this study, based on 179 expression profiles from 13 immune cell datasets, we first applied the WGCNA algorithm to filter out gene modules that were substantially linked to CD8^+^ T cells and obtained 446 genes. Pathway enrichment analysis revealed that 446 genes were intimately linked to immune function as well as associated pathways. Of the 446 genes, 111 genes were strongly linked to prognosis in the TCGA and GSE65858 datasets and were included in subsequent analysis. Based on 111 prognosis-related genes, we divided 499 HNSCC specimens into three subtypes (IC1, IC2, and IC3) in the TCGA dataset. The prognostic analysis of TCGA and GSE65858 revealed that the prognosis of patients was poor in the IC1 group and better in the IC3 group. In terms of immune molecules and functions among molecular subgroups, considerable variations were observed in the expression of chemokines, immune checkpoint genes, immune T cell lysis, and immune cell scores. It was noteworthy that the IFN-*γ* score, immune T cell lytic activity, immune checkpoint genes, and immune microenvironment infiltration levels of the IC3 group were remarkably elevated in contrast with those of the IC1 and IC2 groups; therefore, patients in the IC3 group exhibited a better prognosis. Lastly, we analysed the differences in drug treatment response among the three subtypes. The IC3 subtype was more sensitive to anti-PD-1. Simultaneously, we also analysed the response degree of different subtypes to traditional chemotherapy drugs such as cisplatin, erlotinib, sorafenib, paclitaxel, and AKT inhibitor VIII and found that, as opposed to other subtypes, the IC1 was exhibited greater sensitivity to the above-mentioned traditional drugs. According to these findings, the molecular subtypes of HNSCC premised on CD8^+^ T cell-related genes distinguished patients at low or high risk and those with different clinical characteristics and exhibited a reliable clinical application prospect.

We constructed the 8-gene signature of HNSCC patients premised on CD8^+^ T cell-related genes, which exhibited significant prognostic value in the TCGA and GEO validation datasets and independently served as a predictor of HNSCC patients' prognoses. The 8-gene signature we constructed included the genes ERP44, AKIRIN2, GRAP, KLRC3, FCGBP, LSR, TNFRSF25, and MT1F. ERP44 is a molecular chaperone protein regulated by pH and belongs to the disulfide isomerase family [26]. ERP44 can not only regulate protein maturation and secretion but also participate in the modulation of calcium as well as redox homeostasis in the endoplasmic reticulum [26, 27]. In nasopharyngeal carcinoma, the interaction between ERP44 and ACLY promotes the malignant phenotype of nasopharyngeal carcinoma cells [28]. In addition, ERP44 inhibits the migratory ability of lung cancer cells through IP3R2 [29]. More importantly, honokiol can promote the apoptosis of oral squamous cell carcinoma cells and exert anti-cancer effects by inhibiting the expression of ERP44 in oral squamous cell carcinoma cells [30]. It further promotes the therapeutic potential of ERP44 as a drug target. AKIRIN2 encodes a new member of the innate immune system [31]. AKIRIN2 and nuclear factor kappa B (NF-kB) work together to participate in the transcription of immune response genes downstream of the toll-like receptor (TLR) signalling pathway [32]. AKIRIN2 is necessary for the growth and metastasis of lung cancer [33] and liver cancer [34] and promotes the angiogenesis of gallbladder cancer through interleukin-6 (IL-6)/signal transducer and activator of transcription 3 (STAT3)/vascular endothelial growth factor A (VEGFA) [35]. In addition, AKIRIN2 participates in the regulation of chemotherapy sensitivity of glioma [36], chronic myelogenous leukaemia [37], and ovarian cancer [38]. GRAP encodes members of the GRB2/Sem5/Drk family [39] and functions as a cytoplasmic signalling protein in the inner ear and hearing [40]. The high expression of GRAP is strongly linked to HNSCC patients' better prognoses. The malignant phenotype of oral squamous cell carcinoma cells is inhibited through the Ras/Erk pathway [41]. KLRC3, which is identified as a natural killer receptor gene, performs an instrumental function in tumorigenesis and aggressiveness of glioblastoma [42]. FCGBP is the Fc fragment connexin of immunoglobulin G. Recent studies have demonstrated that FCGBP has a similar mucin-like structure, and its expression is reduced in many solid tumours such as gallbladder cancer [43], thyroid cancer [44], and colon cancer [45], suggesting that it is associated with tumour incidence and progression. LSR encodes lipolytically activated lipoprotein receptors, which bind to chylo particles, very-low-density lipoprotein (VLDL), and low-density lipoprotein (LDL) in the presence of free fatty acids and promote uptake by cells [46]. Studies have suggested that LSR enhances the invasive and metastatic capacities of lung cancer cells [47]. In addition, antibody therapy targeting LSR inhibits the growth of epithelial ovarian tumours by inhibiting lipid absorption [48], further indicating the potential of LSR as a therapeutic target. The protein encoded by TNFRSF25 is the TNFSF12/APO3L/TWEAK receptor, which directly interacts with the adaptor TRADD to mediate the activation of NF-*κ*B and induce cell apoptosis [49]. MT1F belongs to the metallothionein family of proteins, which can bind to different heavy metals. Both glucocorticoids and heavy metals are responsible for the transcriptional regulation of these proteins. MT1F acts as a tumour suppressor in colon cancer [50], gastric cancer [51], and liver cancer [52] and as an oncogene in lung cancer [53] and breast cancer [54]. In conclusion, this research identified for the first time ERP44, AKIRIN2, KLRC3, FCGBP, LSR, TNFRSF25, and MT1F as prognostic biomarkers for patients with HNSCC; however, their selective impacts and possible modulatory processes warrant additional research. Furthermore, some pathways related to immune function and metabolism were decreased with an increase in risk scores. The same results were verified in other articles, for example, the molecular mechanism of prostate cancer and its relationship with immune cell infiltration has been found that the progression of hormone-sensitive prostate cancer to castration-resistant prostate cancer may be related to arachidonic acid metabolism [55].

HNSCC is a malignant tumour with a high risk of recurrence and limited treatment options. However, the quality of a patient's life after treatment deteriorates sharply [56]. Patients who have HNSCC that has recurred or metastasized have fewer treatment choices available, and their prognoses are dismal, with a median OS time of less than one year [57]. With continuous research and the development of immune checkpoint inhibitors in cancer treatment, the prognosis of patients with recurrent or metastatic HNSCC has been improved to some extent. However, some patients cannot benefit from these inhibitors. Some studies have found that the disease may progress after PD-1/PD-L1 inhibitor treatment [58]. PD-1/PD-L1 inhibitor immunotherapy is expensive and has a certain risk of toxicity. Individualised medication can effectively reduce the occurrence of adverse events. Therefore, it is crucial to identify stable and efficient biomarkers. In this study, we constructed the 8-gene signature depending on CD8^+^ T cells-related genes in HNSCC patients undergoing immunotherapeutic treatments. There was a correlation between a greater risk score and a lower chance of survival. The ROC curve demonstrated that risk score had a higher AUC value, and a combination of risk score, TMB, and NEO predicted the immunotherapy response. These results indicate that the 8-gene signature can predict the response to immunotherapy and the efficacy of immunotherapy in HNSCC patients.

This quality study has certain shortcomings. First, our study employed retrospective samples, and prospective samples require to be verified. Furthermore, we only compared changes in the mRNA levels of the 8-gene signature in HNSCC tissues, and changes in the protein levels remain unclear. Lastly, we only analysed the prognostic significance of the 8-gene signature, and we expect to carry out the further cell and animal experiments to investigate the functions of related genes and their regulatory effects on CD8^+^ T cells. Besides, the 8-gene signature needs to be further validated in multicenter clinical trials and larger prospective studies, which would provide a better index for immunotherapy of HNSCC patients.

Using genes associated with CD8^+^ T cells, we generated a molecular subtype of HNSCC in this research. Between the three subtypes, there were significant variations in the patient prognosis, clinical features, immunological molecules, and therapy responsiveness. In addition, the 8-gene signature we constructed exhibited optimal performance in anticipating the prognosis and immunotherapy responsiveness of HNSCC patients and had potential clinical application value.

## Figures and Tables

**Table 1:** Sample information.

Clinical features	TCGA-HNSC	GSE65858
OS		
0	282	176
1	217	94

T stage		
T1	34	35
T2	142	80
T3	132	58
T4	180	97
TX	11	

N stage		
N0	240	94
N1	81	32
N2	152	132
N3	7	12
NX	19	

M stage		
M0	474	
M1	5	
MX	20	

Stage		
I	25	18
II	80	37
III	90	37
IV	304	178

Gender		
Male	366	223
Female	133	47

Age		
≤60	244	153
＞60	255	117
NA		

Smoking		
1	111	
2	169	
3	72	
4	135	
5	2	
7	10	

Alcohol		
YES	331	
NO	157	
NA	11	

HPV		
Negative	64	
Positive	19	
NA	416	

Grade		
G1	61	
G2	298	
G3	119	
G4	2	
GX	19	

**Table 2:** The clinical information of the TCGA training set and validation set.

Clinical features	TCGA-HNSC train	TCGA-HNSC test	*P*
OS			
0	192	90	0.3515
1	157	60	

T stage			
T1	25	9	0.8781
T2	97	45	
T3	90	42	
T4	128	52	

N stage			
N0	165	75	0.3787
N1	55	26	
N2	110	42	
N3	3	4	

M stage			
M0	330	144	0.9894
M1	4	1	

Stage			
I	17	8	0.9973
II	56	24	
III	63	27	
IV	213	91	

Gender			
Male	248	118	0.0986
Female	101	32	

Age			
≤60	167	77	0.538
＞60	182	73	

Smoking			
1	81	30	0.7446
2	115	54	
3	48	24	
4	94	41	
5	2	0	

Alcohol			
YES	227	104	0.6082
NO	112	45	

HPV			
Negative	38	26	0.9769
Positive	12	7	

Grade			
G1	37	24	0.3393
G2	211	87	
G3	83	36	
G4	2	0	

## Data Availability

Upon a reasonable request, the corresponding author of this research will make the data that were used to corroborate the conclusions of this study accessible to the requesting party.

## Supplementary Materials

Supplementary Materials Supplementary Figure 1: (a) PCA analysis before eliminating the batch effect; (b) PCA analysis after eliminating the batch effect. Supplementary Figure 2: (a) BP annotation of the pink module gene; (b) MF annotation of the pink module gene; (c) CC annotation of the pink module gene; (d) KEGG annotation of the pink module gene. Supplementary Figure 3: comparison of the distribution of different clinical characteristics among the three molecular subtypes in the TCGA dataset. Supplementary Figure 4: (a) risk score, survival time, survival status, and the expression of eight genes in the TCGA training set; (b) ROC curve and AUC of the 8-gene signature; (c) KM survival curve distribution of the 8-gene signature in the validation set. Supplementary Figure 5: (a) risk score, survival time, survival status, and the expression of eight genes in all TCGA datasets; (b) ROC curve and AUC of the 8-gene signature; (c) KM survival curve distribution of the 8-gene signature in all datasets. Supplementary Figure 6: (a) risk score, survival time, survival status, and the expression of eight genes in the independent verification dataset GSE65858; (b) ROC curve and AUC of the 8-gene signature; (c) KM survival curve distribution of the 8-gene signature in the independent verification dataset.
