# Supplementary material for: Systematic Analysis of Molecular Subtypes and Immune Prediction Based on CD8 T Cell Pattern Genes Based on Head and Neck Cancer
Source: J Oncol. 2022 Aug 25;2022:1500493. doi: 10.1155/2022/1500493 (PMC9436594; doi:10.1155/2022/1500493)
Supplement: Supplementary Materials — Supplementary Figure 1: (a) PCA analysis before eliminating the batch effect; (b) PCA analysis after eliminating the batch effect. Supplementary Figure 2: (a) BP annotation of the pink module gene; (b) MF annotation of the pink module gene; (c) CC annotation of the pink module gene; (d) KEGG annotation of the pink module gene. Supplementary Figure 3: comparison of the distribution of different clinical characteristics among the three molecular subtypes in the TCGA dataset. Supplementary Figure 4: (a) risk score, survival time, survival status, and the expression of eight genes in the TCGA training set; (b) ROC curve and AUC of the 8-gene signature; (c) KM survival curve distribution of the 8-gene signature in the validation set. Supplementary Figure 5: (a) risk score, survival time, survival status, and the expression of eight genes in all TCGA datasets; (b) ROC curve and AUC of the 8-gene signature; (c) KM survival curve distribution of the 8-gene signature in all datasets. Supplementary Figure 6: (a) risk score, survival time, survival status, and the expression of eight genes in the independent verification dataset GSE65858; (b) ROC curve and AUC of the 8-gene signature; (c) KM survival curve distribution of the 8-gene signature in the independent verification dataset. [file 1500493.f1.docx]

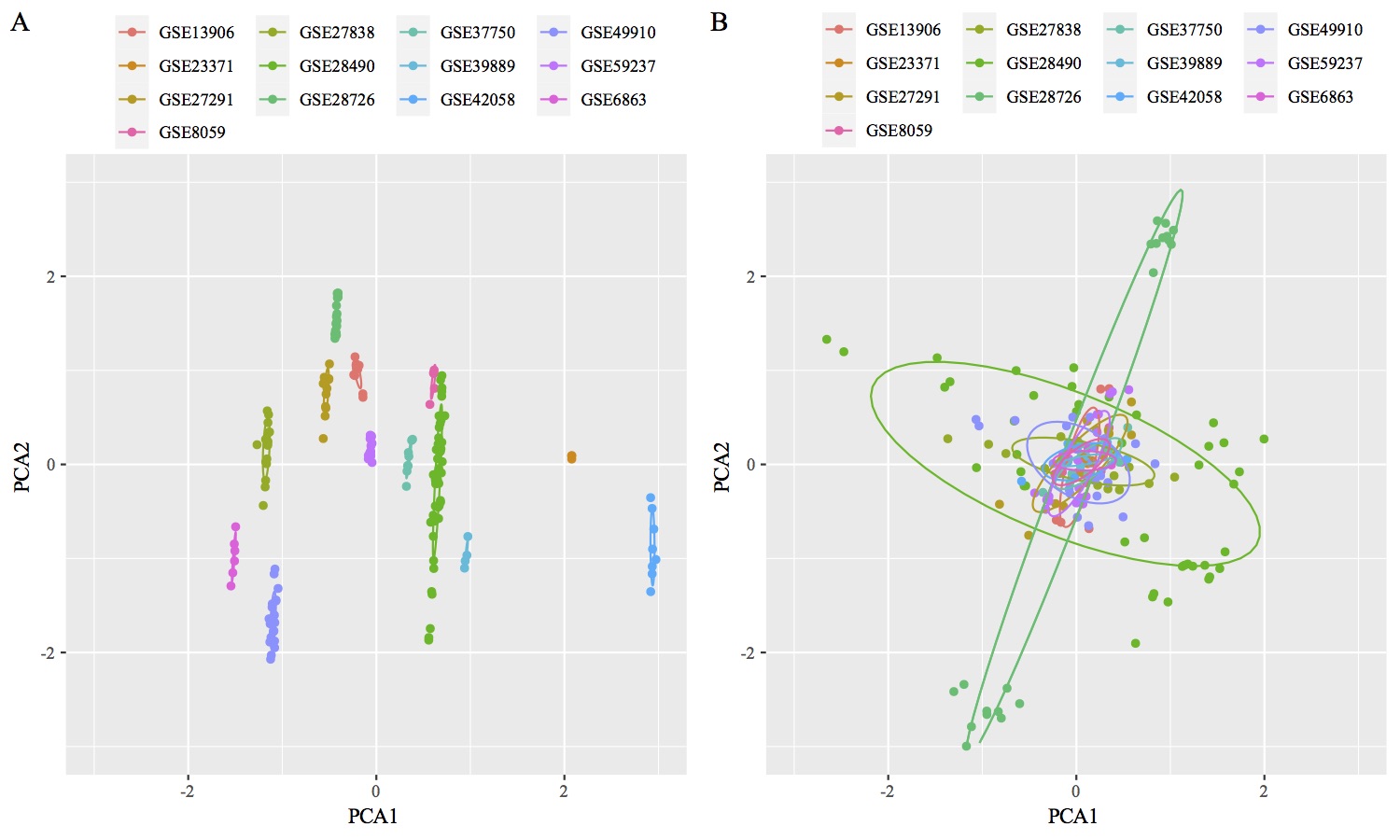


Supplement Fig. 1 (A) PCA analysis before eliminating the batch effect; (B) PCA analysis after eliminating the batch effect.


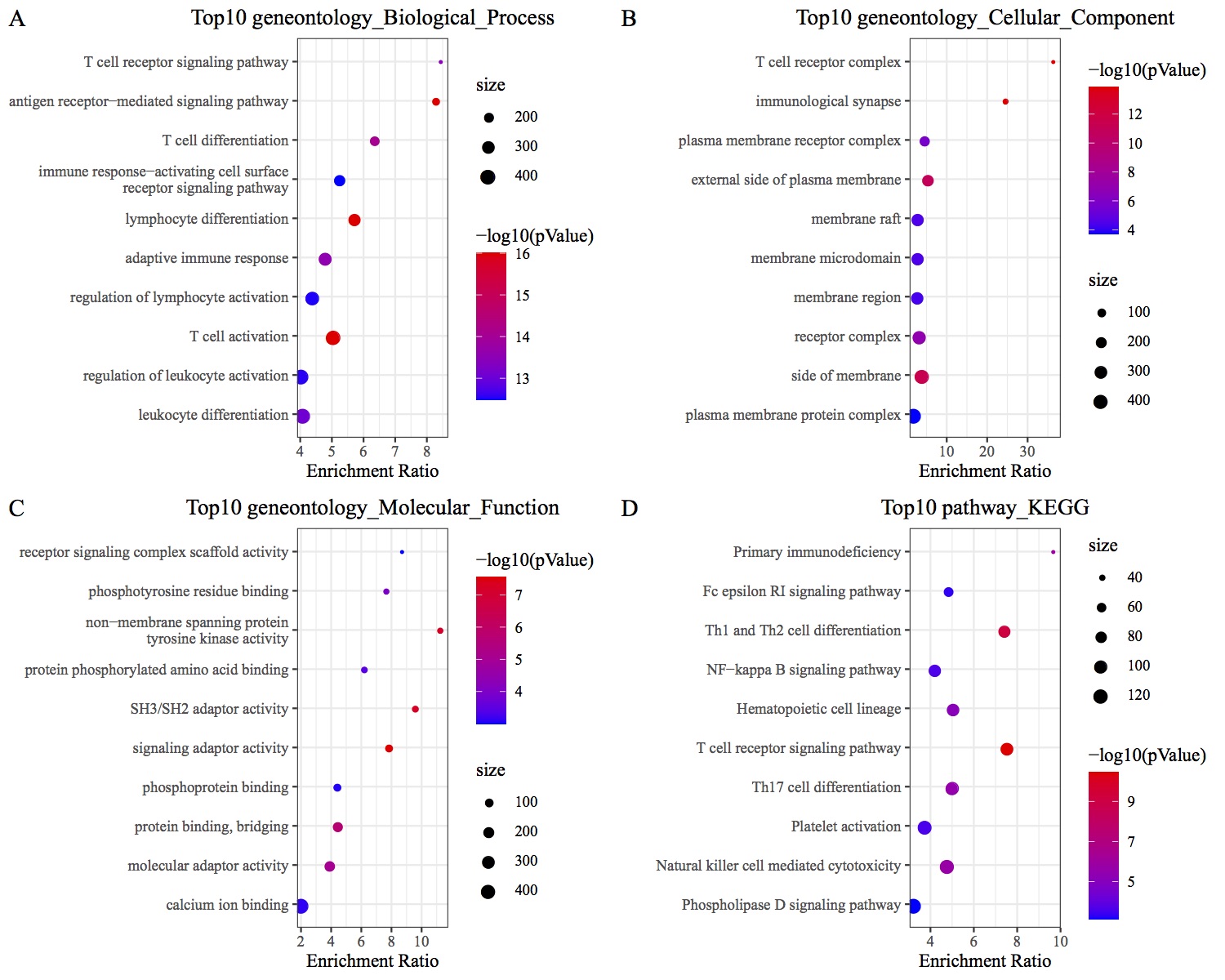


Supplement Fig. 2 (A) BP annotation of the pink module gene; (B) MF annotation of the pink module gene; (C) CC annotation of the pink module gene; (D) KEGG annotation of the pink module gene.


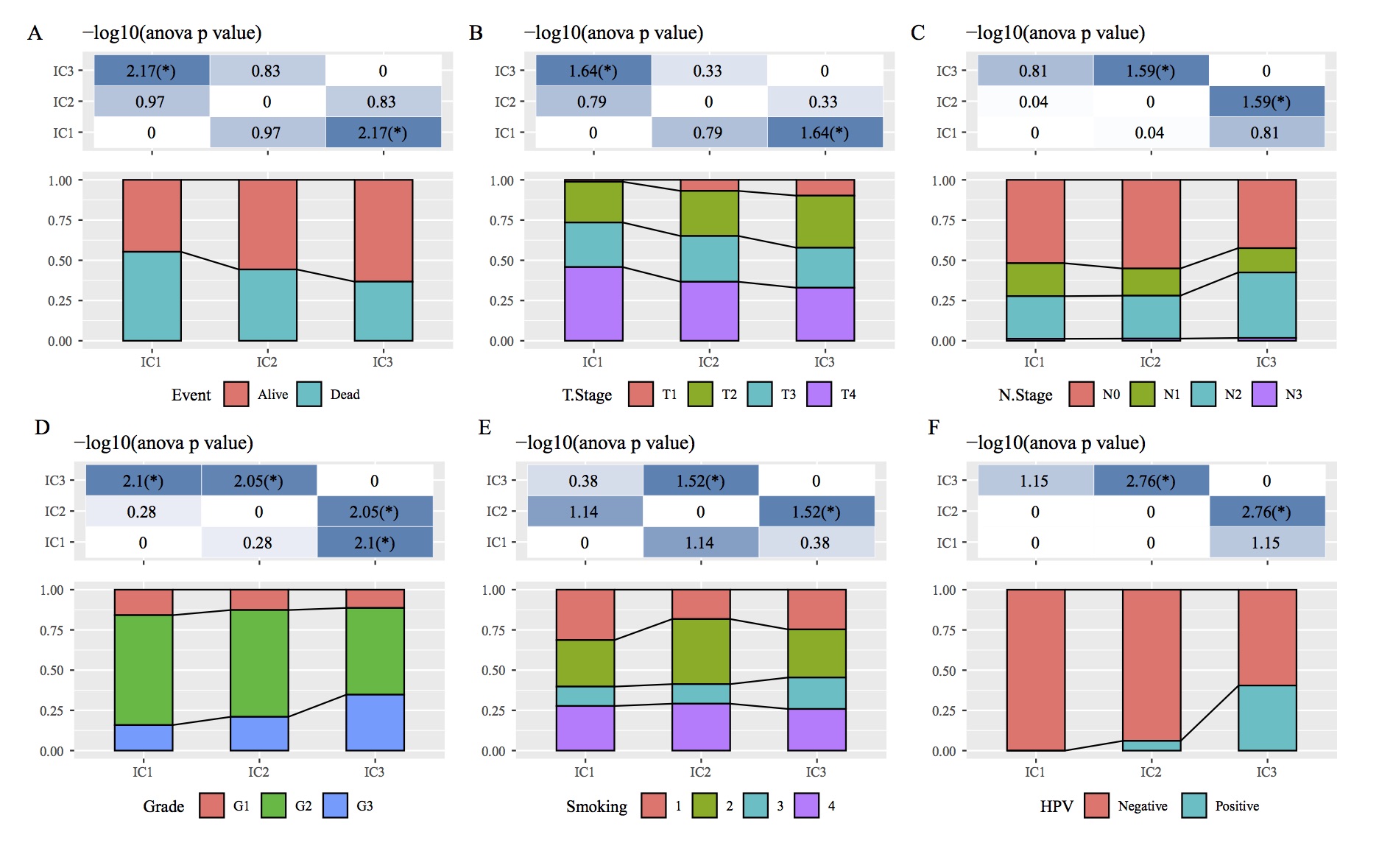


Supplement Fig. 3 Comparison of the distribution of different clinical characteristics among the three molecular subtypes in the TCGA dataset.


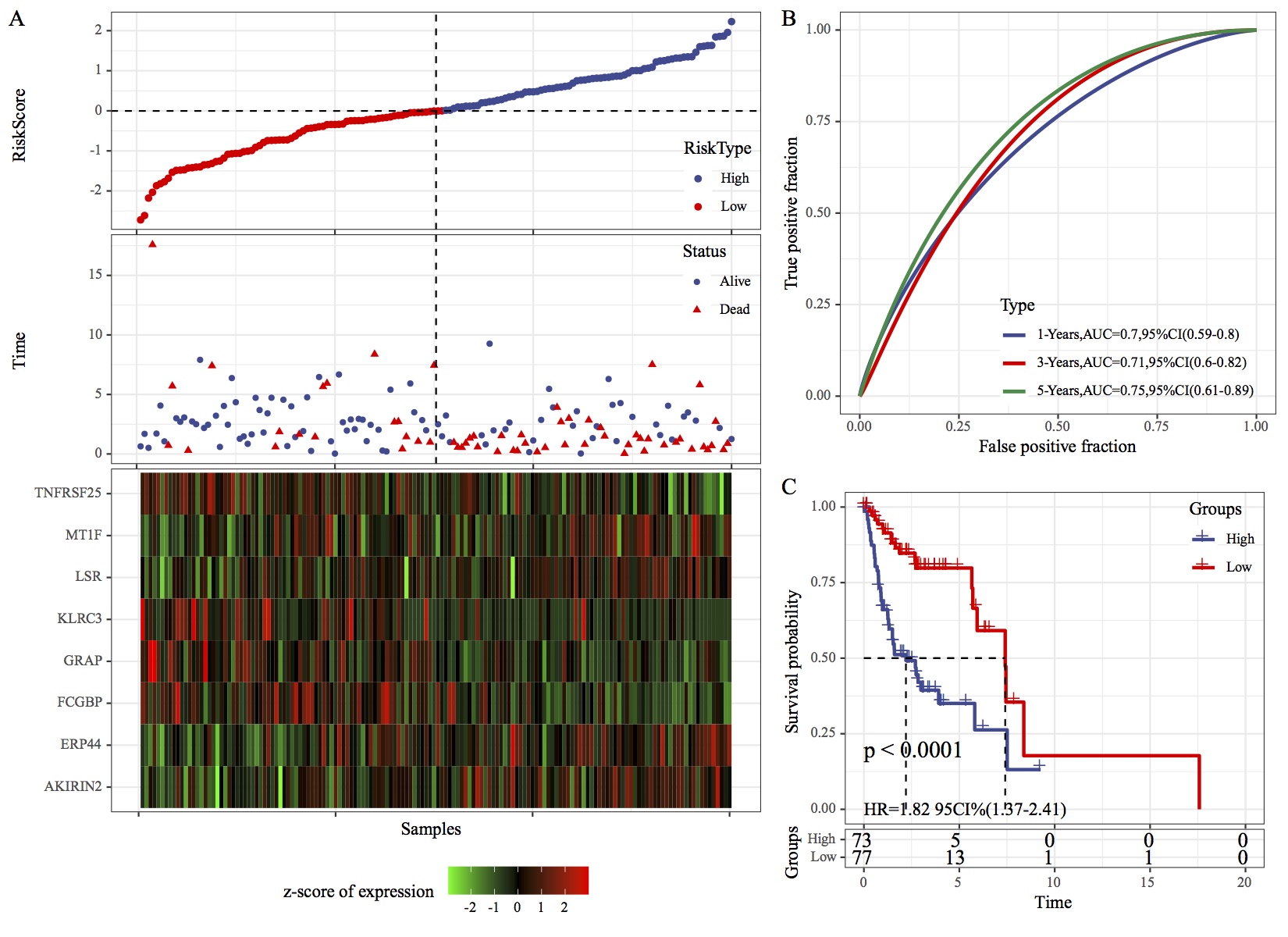


Supplement Fig. 4 (A) Risk score, survival time, survival status and the expression of eight genes in the TCGA training set; (B) ROC curve and AUC of the 8-gene signature; (C) KM survival curve distribution of the 8-gene signature in the validation set.


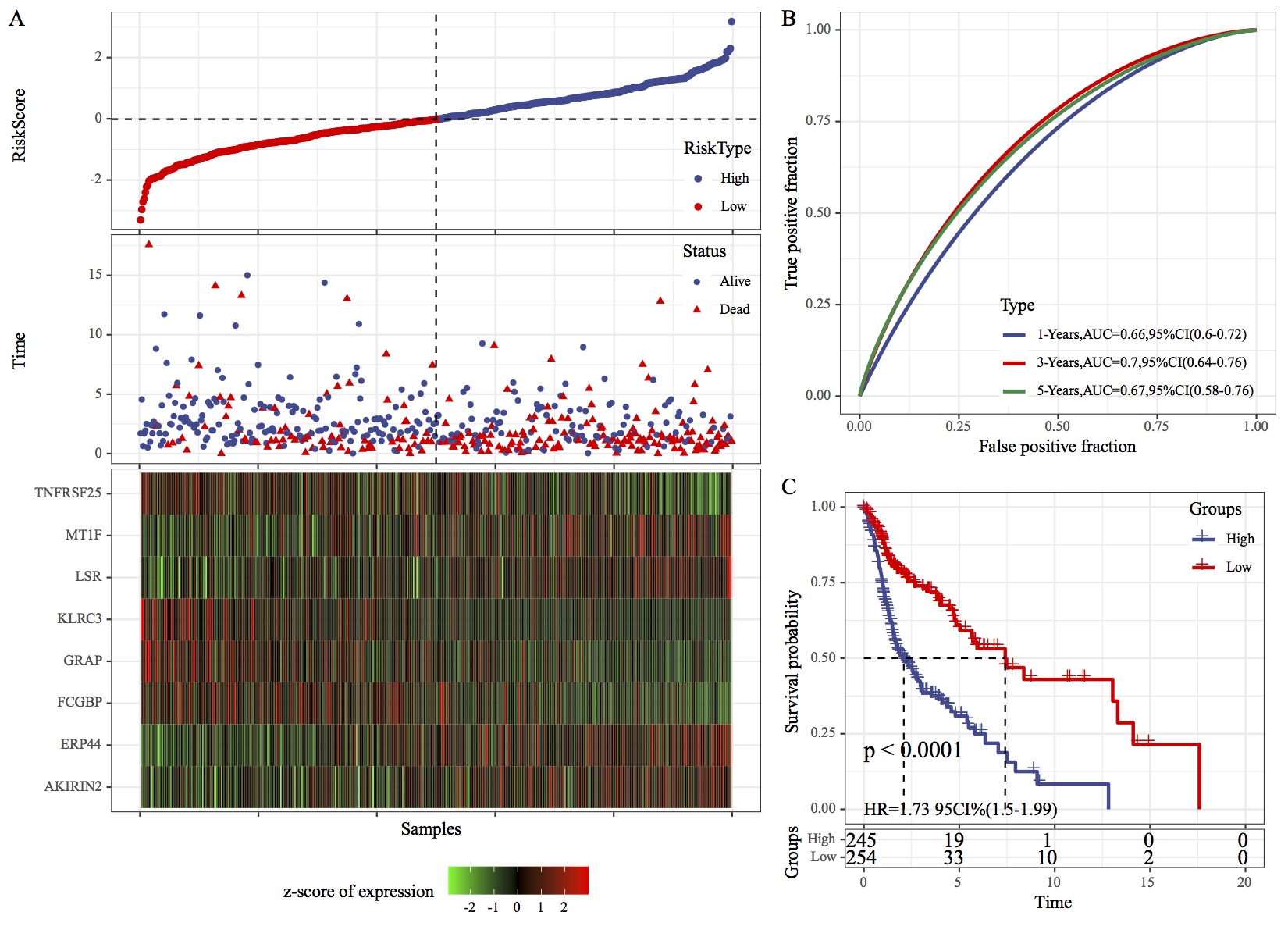


Supplement Fig. 5 (A) Risk score, survival time, survival status and the expression of eight genes in all TCGA datasets; (B) ROC curve and AUC of the 8-gene signature; (C) KM survival curve distribution of the 8-gene signature in all datasets.


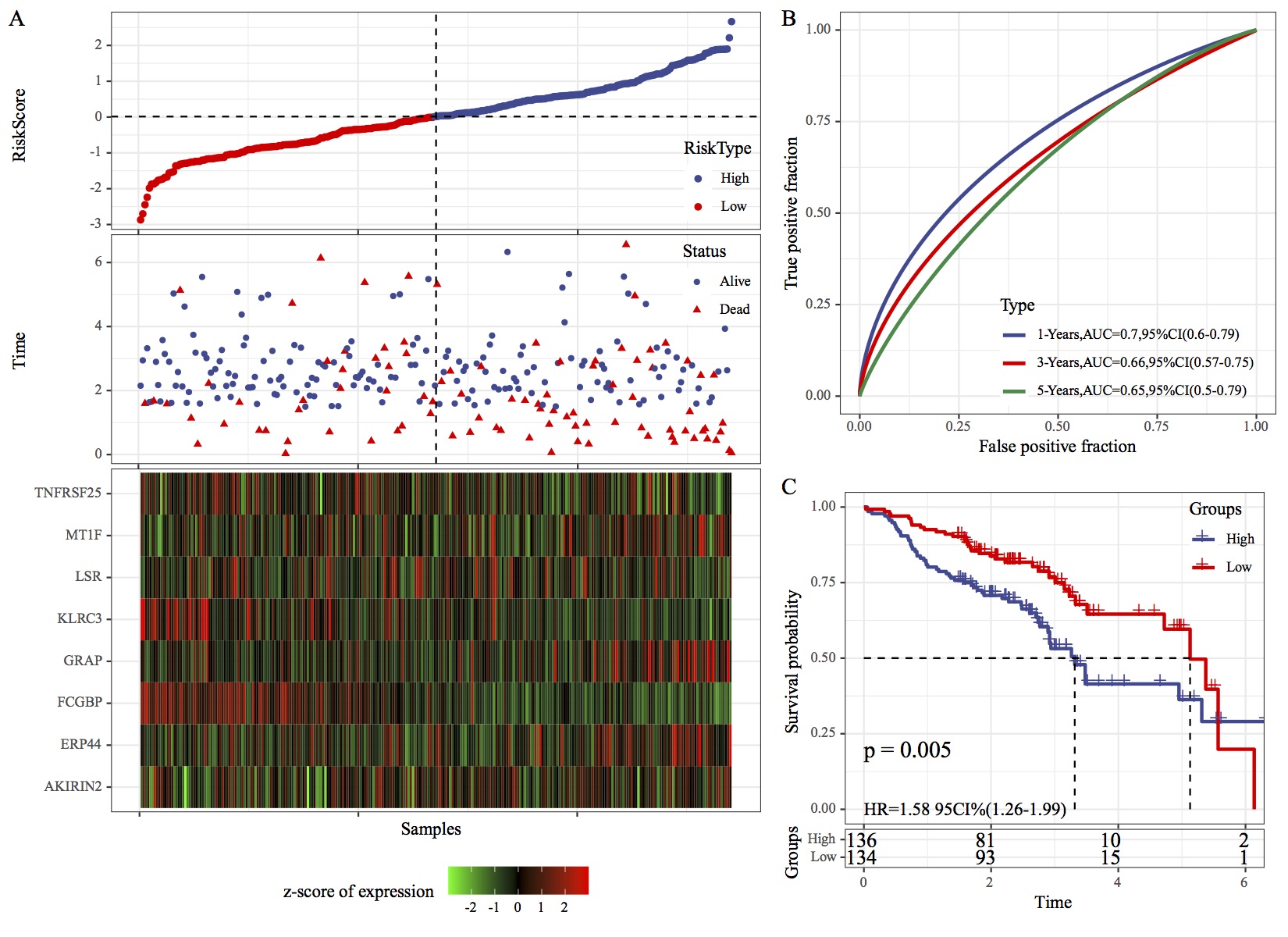


Supplement Fig. 6 (A) Risk score, survival time, survival status and the expression of eight genes in the independent verification dataset GSE65858; (B) ROC curve and AUC of the 8-gene signature; (C) KM survival curve distribution of the 8-gene signature in the independent verification dataset.

Supplementary table 1. The chip expression data of 14 immune cells

B cells CD4 T cells CD8 T cells Dendritic cells Eosinophils Gamma-delta T cells Immature dendritic cells Lymphocyte Monocytes Myeloid Dendritic Cells Natural Killer cells Natural killer T cells Neutrophils Plasmacytoid dendritic cells

GSE13906 0 0 0 0 0 8 0 2 0 0 0 0 0 0

GSE23371 0 0 0 0 0 0 3 0 0 0 0 0 0 0

GSE27291 0 0 0 0 0 12 0 0 0 0 0 0 0 0

GSE27838 0 0 0 0 0 0 0 0 0 0 16 0 0 0

GSE28490 5 5 5 0 4 0 0 0 10 5 5 0 3 5

GSE28726 0 8 0 0 0 0 0 0 0 0 0 12 0 0

GSE37750 0 0 0 0 0 0 0 0 0 0 0 0 0 8

GSE39889 0 0 0 0 0 0 0 0 0 0 0 0 4 0

GSE42058 0 0 0 0 0 0 0 0 0 8 0 0 0 0

GSE49910 4 3 6 0 0 0 0 0 6 0 0 0 3 0

GSE59237 0 0 0 18 0 0 0 0 0 0 0 0 0 0

GSE6863 0 0 0 0 0 0 6 0 0 0 0 0 0 0

GSE8059 0 0 1 0 0 0 0 0 0 0 4 0 0 0

**Supplementary table 2. The univariate survival analysis of CD8 T cell-related genes from the TCGA**

p.value HR Low 95%CI High 95%CI

MYOM2 0.034947369 1.116565022 1.007820349 1.237043337

EOMES 0.00303769 0.775370534 0.655305916 0.917433295

CD96 0.003444629 0.814160819 0.709380713 0.934417623

ZNF662 0.00225422 0.694816363 0.550052682 0.877679166

ZAP70 6.43E-06 0.73964944 0.648829211 0.843182282

ERP44 0.000320415 1.719899163 1.280039208 2.310908221

AKIRIN2 0.013332207 1.436511521 1.078260535 1.91379104

RHOH 0.019772439 0.827835594 0.706217868 0.970397101

ZNF540 0.013970321 0.572936365 0.367474225 0.89327647

KLRB1 0.000972988 0.79336331 0.691406675 0.910354737

NAP1L2 0.007532141 1.153230917 1.038736707 1.280345191

LAT 0.000602897 0.389784787 0.227528501 0.667750105

SFI1 0.029471322 0.798215854 0.651634135 0.97777037

PBX4 0.009464759 0.783302618 0.651343145 0.941996543

GIMAP7 0.002846001 0.851797402 0.766616146 0.946443429

ICOS 0.001369856 0.807541947 0.708469641 0.920468513

TCEA3 0.023376772 0.889732633 0.804258107 0.984291176

HOOK1 0.004069978 0.82951775 0.730201003 0.942342855

GZMH 0.030100632 0.910392487 0.836339534 0.991002393

CD3D 0.000457744 0.846116721 0.770623571 0.92900546

PHLDB2 0.013368858 1.163769696 1.032003321 1.312360028

UBASH3A 0.00041922 0.743991209 0.631264561 0.876847764

ZNF831 8.14E-05 0.541360298 0.398944916 0.734615132

FCRL3 9.80E-05 0.619557929 0.486935036 0.788302338

CD28 0.001287731 0.76031844 0.643472647 0.898381823

SIRPG 0.000861302 0.815369912 0.723129511 0.919376243

CD7 0.001261374 0.835510198 0.749054044 0.931945162

GRAP 0.00191226 0.591696167 0.424788697 0.82418472

CTSW 0.004073453 0.860287238 0.776333153 0.953320271

TXK 0.000504767 0.525033825 0.36518555 0.754850561

NKG7 0.025539386 0.911293447 0.839946274 0.988701031

LAIR2 0.018802695 0.790166711 0.649220609 0.961712278

KLRC3 0.006346765 0.251648507 0.09343166 0.677789214

FCGBP 0.001149857 0.864602168 0.791998816 0.943861144

CD2 0.001750372 0.865975968 0.79134887 0.947640674

CD247 0.001509325 0.816072484 0.719782925 0.925243259

TRAT1 0.003726148 0.77507787 0.652486147 0.920702618

GZMM 3.99E-05 0.784036075 0.698108851 0.880539712

KLRG1 0.001024207 0.60937073 0.453404683 0.818987321

LRRC8A 0.036190197 1.190529307 1.011287461 1.401540199

SAMD3 0.001767899 0.492334728 0.315757542 0.767656991

CD8A 0.01114624 0.890229323 0.813778286 0.973862612

DENND2D 0.00310395 0.74487468 0.612779723 0.905444922

PASK 0.004807073 0.757723553 0.624825451 0.918888598

ABCB1 0.000858251 0.672669828 0.532782649 0.849285723

EIF5A2 0.003014406 1.262738507 1.082356751 1.473182051

LAX1 0.000644699 0.788648672 0.688099879 0.903890187

PRSS23 0.011245025 1.145354872 1.03126368 1.272068248

CXorf65 0.002372883 0.616932289 0.451812457 0.84239698

MIAT 0.008051137 0.831580354 0.725542435 0.953115698

KLRF1 0.003710805 0.674236579 0.516636495 0.879912606

ST8SIA1 0.023634986 0.767269821 0.609962611 0.965146006

BNIP3 0.004943273 1.224974992 1.063345259 1.411172635

FAM169A 0.01103498 1.275866044 1.057335376 1.539562751

CD3E 0.000619132 0.843390656 0.765023883 0.929785088

KLRD1 0.012304151 0.674029991 0.494929351 0.917941982

SPOCK2 0.000168455 0.825505207 0.747022143 0.912233799

SH2D1A 0.001426056 0.805954392 0.70588606 0.920208684

ZNF101 0.003193441 0.700325838 0.552669261 0.887431804

GPRASP1 0.033528909 0.752543698 0.579014664 0.978078886

BTK 0.016704369 0.831190865 0.714391993 0.967085663

PRF1 0.010451132 0.880614786 0.798955026 0.970620843

NCALD 0.015549528 0.851745769 0.747914615 0.969991549

GZMK 0.005206701 0.86949064 0.788241352 0.959114833

LSR 0.040476071 1.211758214 1.008368221 1.456172396

CD3G 0.001994546 0.816821396 0.718470256 0.928635789

ITM2A 0.023765747 0.881180834 0.78966352 0.983304461

CD27 5.86E-05 0.825287404 0.751497972 0.906322206

LCK 0.001462116 0.831562207 0.742258487 0.931610369

TNFRSF25 0.000969168 0.779918974 0.67285507 0.904018759

LY9 0.00101112 0.668731239 0.526097473 0.850035389

SLAMF6 0.000378297 0.812785202 0.725003341 0.911195505

PYHIN1 0.007635298 0.781603473 0.652177748 0.936713943

GAPT 0.004294735 0.699960622 0.547949969 0.89414162

CCL5 0.02586909 0.910628326 0.838641712 0.988794065

TRPC1 0.021123 1.194750924 1.027045919 1.389840263

BAG3 0.029250701 1.306656635 1.027375032 1.661858143

TIGIT 0.001647724 0.812050156 0.713307013 0.924462319

IKZF3 0.001624811 0.838338072 0.751261595 0.935507322

MT1F 0.009388165 1.193697369 1.044419579 1.364311276

CD5 6.45E-05 0.799050764 0.715792268 0.891993602

GZMB 0.007796567 0.886490023 0.811202709 0.968764716

GRAP2 0.003094586 0.747515262 0.616433429 0.906471064

SLA2 0.00600047 0.83649286 0.736469626 0.950100697

PLCG2 0.049596534 0.829971999 0.689080008 0.999671317

CD6 4.31E-05 0.760823076 0.667414954 0.86730414

TBX21 0.006821317 0.776845136 0.646974308 0.932785673

CST7 0.002231238 0.839746743 0.750803372 0.939226727

CD226 0.002277754 0.566524743 0.393285058 0.81607546

ITK 0.0006179 0.746165892 0.631009796 0.882337393

**Supplementary table 3. The univariate survival analysis of CD8 T cell-related genes from GSE65858 datasets**

p.value HR Low 95%CI High 95%CI

STRN4 0.011001967 0.134138709 0.028512714 0.631058584

EPHX2 0.02338976 0.622409126 0.413087705 0.93779872

SFI1 0.041366901 0.180407058 0.034803941 0.935144291

TCEA3 0.008496319 0.689152682 0.522275624 0.909350155

BHLHB9 0.04364739 0.384229863 0.151712487 0.973107691

SBK1 0.039775337 0.621531757 0.394985482 0.978014995

ABLIM1 0.002789096 0.506326071 0.324106155 0.790994204

FCGBP 0.008134908 0.710603849 0.551748862 0.915195056

ZNF571 0.012493041 0.044243376 0.003831082 0.510946007

SLC25A23 0.003876512 0.578126854 0.398584568 0.838543902

STX7 0.038095396 3.947544103 1.078303854 14.45149656

STX2 0.010514828 2.164382466 1.197954482 3.910458643

RBM11 0.004252801 0.038585228 0.004142642 0.359388945

PRSS23 0.044592602 1.355590765 1.007375692 1.824171794

F2R 0.044552868 3.002443812 1.027116937 8.776672376

DLG5 0.013794636 0.307739878 0.120455831 0.786212106

MATK 0.008329411 3.496718528 1.379745695 8.861807295

UBLCP1 0.026317112 2.195864144 1.097026291 4.395354404

AGMAT 0.006899329 0.010798966 0.000404268 0.288466343

ACSL6 0.031324937 34.85238545 1.374748438 883.571669

INPP4B 0.038404692 1.581854636 1.024786903 2.441740894

KRTCAP3 0.013457624 0.644534291 0.454955044 0.913110991

TGFBR3 0.003405994 0.58207546 0.405215971 0.836126574

MICAL1 0.005570472 1.844073465 1.196357492 2.842467213

LCN2 0.006280628 0.811737211 0.698951835 0.94272204

**Supplementary table 4. The results of univariate Cox analysis**

p.value HR Low 95%CI High 95%CI

EOMES 0.015561194 0.785099186 0.645342319 0.955122133

CD96 0.026598388 0.833860431 0.710144028 0.979129855

ZNF662 0.015124596 0.720745811 0.553406597 0.938685095

ZAP70 0.002276228 0.791849387 0.681621683 0.919902443

ERP44 0.001699 1.759250929 1.236276766 2.503455469

AKIRIN2 0.020007549 1.477889586 1.063429623 2.053880746

KLRB1 0.011345363 0.814116013 0.694300107 0.954608644

NAP1L2 0.025615692 1.14707827 1.016864156 1.293966898

LAT 0.011072037 0.454700111 0.24754582 0.835207767

GIMAP7 0.026174806 0.870248292 0.769914726 0.983657104

ICOS 0.01933034 0.834823893 0.717625879 0.971161928

CD3D 0.004874598 0.855046509 0.766726669 0.95353998

PHLDB2 0.006329756 1.209207603 1.055054069 1.385884448

RAP1B 0.049596491 1.278537277 1.000433574 1.633949131

UBASH3A 0.004153207 0.757265116 0.626149101 0.915836908

ZNF831 0.002177718 0.574274431 0.4027889 0.818769142

FCRL3 0.002125573 0.65249843 0.496916481 0.856792273

CD28 0.015888299 0.783791555 0.642999031 0.955412328

SIRPG 0.009495162 0.832360423 0.724591926 0.95615732

CD7 0.011781446 0.847102914 0.744486691 0.963863229

GRAP 0.013531952 0.601650702 0.40198866 0.900482036

CTSW 0.022788113 0.870107216 0.771895216 0.98081521

TXK 0.018268831 0.615340735 0.411137276 0.920967867

NKG7 0.039141963 0.90553033 0.824047686 0.995070057

KLRC3 0.028713979 0.301805323 0.103168608 0.882889233

FCGBP 0.015270701 0.883026117 0.79858972 0.976390134

CD2 0.010854201 0.873334853 0.786909299 0.96925245

CD247 0.014046727 0.833042568 0.720043721 0.963774698

TRAT1 0.040935823 0.816056725 0.671548856 0.991660656

GZMM 0.000649085 0.792349815 0.693140709 0.905758702

KLRG1 0.003315548 0.598248644 0.424599871 0.842914623

SAMD3 0.027713238 0.552589594 0.325873439 0.937036356

CD8A 0.031738979 0.891628849 0.803012658 0.99002425

DENND2D 0.020225542 0.762928052 0.607149548 0.958675196

PASK 0.043713196 0.79922812 0.642814714 0.993700944

ABCB1 0.025787772 0.7357224 0.561740816 0.963589319

EIF5A2 0.0086583 1.266940254 1.061794673 1.511721284

LAX1 0.023183684 0.832056762 0.709942807 0.975174969

PRSS23 0.041137924 1.130113285 1.004934061 1.27088541

CXorf65 0.011202253 0.637299834 0.449934258 0.902689828

KLRF1 0.022860755 0.704841593 0.521509565 0.952622359

CD3E 0.005217035 0.850200733 0.758698811 0.95273813

SPOCK2 0.006775661 0.852411842 0.759363516 0.956861809

SH2D1A 0.012291319 0.820622306 0.702961496 0.957977033

PRF1 0.026827981 0.880218191 0.786210215 0.985466799

GZMK 0.026011422 0.879460952 0.785418055 0.98476418

LSR 0.015880454 1.297445101 1.049979626 1.603234719

ITGA6 0.029559326 1.153908265 1.014310787 1.312718254

CD3G 0.016012375 0.832922978 0.717792735 0.966519516

CD27 0.001402407 0.835565573 0.74835395 0.932940659

LCK 0.025308144 0.857168053 0.748880455 0.981113964

TNFRSF25 0.027502629 0.822374249 0.691119976 0.978555721

LY9 0.0065159 0.672513608 0.505330157 0.895008039

SLAMF6 0.006632999 0.831305146 0.727494326 0.949929396

GAPT 0.02778204 0.724430988 0.543612267 0.965394433

BAG3 0.011613509 1.434705674 1.083966826 1.898932995

TIGIT 0.017578024 0.831339611 0.713770808 0.968273766

IKZF3 0.007166146 0.839208645 0.738551176 0.953584766

MT1F 0.027611998 1.187288741 1.019102166 1.383231831

CD5 0.004900614 0.832525741 0.732730601 0.945912602

GZMB 0.026536835 0.888603337 0.800549982 0.986341776

GRAP2 0.038304754 0.787165287 0.627670525 0.987188603

SLA2 0.040381721 0.856789817 0.739079587 0.993247281

CD6 0.002488532 0.792649714 0.681848804 0.921455849

TBX21 0.012027228 0.756071818 0.607838631 0.94045453

CST7 0.021283553 0.858557512 0.754053057 0.977545273

CD226 0.02557861 0.608976277 0.394003673 0.941240225

ITK 0.019365165 0.791782243 0.651060938 0.962919265
